# A workflow to process 3D+time microscopy images of developing organisms and reconstruct their cell lineage

**DOI:** 10.1038/ncomms9674

**Published:** 2016-02-25

**Authors:** Emmanuel Faure, Thierry Savy, Barbara Rizzi, Camilo Melani, Olga Stašová, Dimitri Fabrèges, Róbert Špir, Mark Hammons, Róbert Čúnderlík, Gaëlle Recher, Benoît Lombardot, Louise Duloquin, Ingrid Colin, Jozef Kollár, Sophie Desnoulez, Pierre Affaticati, Benoît Maury, Adeline Boyreau, Jean-Yves Nief, Pascal Calvat, Philippe Vernier, Monique Frain, Georges Lutfalla, Yannick Kergosien, Pierre Suret, Mariana Remešíková, René Doursat, Alessandro Sarti, Karol Mikula, Nadine Peyriéras, Paul Bourgine

**Affiliations:** 1 Complex Systems Institute Paris Ile-de-France (ISC-PIF, UPS3611), CNRS, 75013 Paris, France; 2 Research Center in Applied Epistemology (CREA, UMR7656), CNRS and Ecole Polytechnique, 75005 Paris, France; 3 Multiscale Dynamics in Animal Morphogenesis (MDAM), Neurobiology & Development (N&D, UPR3294), CNRS, 91198 Gif-sur-Yvette, France; 4 BioEmergences Laboratory (USR3695), CNRS, Université Paris-Saclay, 91198 Gif-sur-Yvette, France; 5 Department of Electronics, Information and Systems, University of Bologna, 40126, Italy; 6 Department of Mathematics and Descriptive Geometry, Slovak University of Technology, 81005 Bratislava, Slovakia; 7 Neurobiology & Development (N&D, UPR3294), CNRS, 91198 Gif-sur-Yvette, France; 8 Computing Center of the National Institute for Nuclear Physics and Particle Physics (CC-IN2P3, USR6402), CNRS, 69100 Villeurbanne, France; 9 Dynamics of Membrane Interactions in Normal and Pathological Cells (DIMNP, UMR5235), CNRS and Université Montpellier 2, 34090 Montpellier, France; 10 Medical Informatics and Knowledge Engineering in e-Health (LIMICS, UMR1142), CNRS and Université Paris 13, 93017 Bobigny, France; 11 Laser, Atomic and Molecular Physics Laboratory (UMR8523), CNRS and Université Lille 1-Science and Technology, 59650 Villeneuve-d'Ascq, France

## Abstract

The quantitative and systematic analysis of embryonic cell dynamics from *in vivo* 3D+time image data sets is a major challenge at the forefront of developmental biology. Despite recent breakthroughs in the microscopy imaging of living systems, producing an accurate cell lineage tree for any developing organism remains a difficult task. We present here the BioEmergences workflow integrating all reconstruction steps from image acquisition and processing to the interactive visualization of reconstructed data. Original mathematical methods and algorithms underlie image filtering, nucleus centre detection, nucleus and membrane segmentation, and cell tracking. They are demonstrated on zebrafish, ascidian and sea urchin embryos with stained nuclei and membranes. Subsequent validation and annotations are carried out using Mov-IT, a custom-made graphical interface. Compared with eight other software tools, our workflow achieved the best lineage score. Delivered in standalone or web service mode, BioEmergences and Mov-IT offer a unique set of tools for *in silico* experimental embryology.

## Abstract (web summary)

Quantitative analysis of embryonic cell dynamics from large data sets remains a major challenge in the field of developmental biology. Here the authors develop software and a workflow to reconstruct cell lineage trees from 3D time lapse imaging data sets from several developing organisms including zebrafish, tunicates and sea urchins.

## Graphical abstract

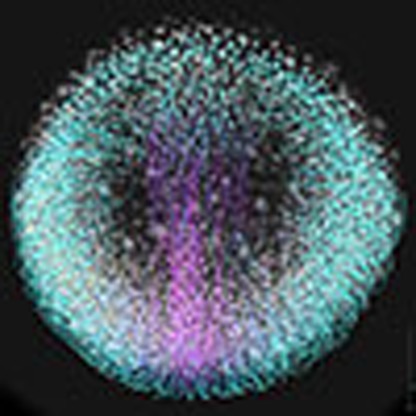

Cells being the necessary level of integration of biological processes^1^, multicellular organization is best described by the cell lineage tree deployed in space and time. Thus, the quantitative investigation of cell behaviour based on lineage branches annotated with relevant measurements at the individual cell level is the indispensable basis for reconstructing the multilevel dynamics of developing organisms^2^. Accurate and precise data about cell positions, trajectories, divisions, nucleus and cell shapes can be derived from the automated processing of 3D+time images. Contributions in the field point to the necessary co-optimization of 4D multimodal imaging techniques and algorithmic image processing workflows^3^,^4^,^5^. Ideally, going from the microscopy data to the interactive visualization of the cell lineage tree and segmented shapes should be automated, easily manageable and fast enough to allow a quantitative comparison of individuals^6^.

In recent years, decisive breakthroughs were made in the microscopy imaging of living systems, thanks to progress in fluorescent protein engineering^7^,^8^,^9^ and microscopy imaging techniques, including multiphoton laser scanning microscopy (MLSM) and selective plane illumination microscopy (SPIM)^10^. Concomitantly, image processing methods for cell segmentation, cell tracking and the analysis of new types of quantitative data have diversified and improved^3^,^4^,^5^,^11^,^12^,^13^. The huge data flow produced by 3D+time imaging of live specimens has also greatly benefited from faster computer hardware and computing grid architectures able to cope with high-dimensional data sets^14^. Finally, computer-aided data analysis and visualization software have completed the toolbox of quantitative developmental biology^15^.

Successful applications are still rare, however, and producing an accurate cell lineage tree for any developing organism remains a difficult challenge. In 2006, the automated reconstruction of the nematode cell lineage from confocal images established the first standard^16^, although it did not yield reliable results beyond the 194-cell stage. Later, reconstructions were attempted on more complex organisms, such as the zebrafish embryo imaged by digital scanned laser light-sheet fluorescence microscopy (DSLM)^17^ or *Drosophila* imaged by MLSM during gastrulation^18^, but they did not provide long-term accurate single-cell tracking either. A concurrent work^4^ on semi-automated cell lineage reconstruction from harmonic generation imaging of non-labelled zebrafish embryos provided six digital specimens with precise nucleus and membrane segmentation, yet was limited to the first 10 divisions of the egg cell. Most recently, Amat *et al*.^5^ delivered a standalone software for the reconstruction of cell lineages from *Drosophila* embryos with fluorescently stained nuclei. Their method, also tested on developing mouse and zebrafish embryos, is well suited for the low background and high temporal resolution of SPIM data. Among all the state-of-the-art algorithmic image processing strategies, whether commercial or open-source software, the latter is the only one offering 3D+time cell tracking with detection of mitotic events to reconstruct the branching dynamics of cell lineage. Altogether, the growing number of solutions available today confirms that the automated reconstruction of cell trajectories and cell shapes, together with their interactive visualization, is at the cutting edge of developmental biology. Obviously, the performance achieved so far in terms of accuracy, scalability and ease of operation leaves plenty of room for improvement. There are still a great number of methods to explore, and more to invent in the fields of image processing and machine vision.

We deliver here an original image processing workflow, BioEmergences, in the form of standalone software. Although optimized for MLSM data and fast cell movements in gastrulating zebrafish embryos, it generally performs well on 3D+time imaging data without heavy requirements in terms of spatial and temporal resolution, or signal-to-noise ratio. In addition to the reconstruction of the cell lineage branching process, the BioEmergences workflow includes segmentation algorithms for cell nucleus and membrane shapes. These are based on the ‘subjective surface' method, which can complete cell contours from heterogeneous fluorescent membrane staining^19^. The standalone version of the workflow can be operated through a graphical user interface, and its output data are connected to Mov-IT, a custom-made interactive visualization software. Alternatively, our web service offers users customized assistance and fast processing on computer clusters or on the European Grid Infrastructure (EGI), together with the possibility to explore a large parameter space for the optimization of results (see Methods to request access).

We demonstrate the reconstruction and analysis of six digital embryos from three different species. All the data obtained, raw and reconstructed, is made available to the community. The BioEmergences workflow is compared with eight other software tools from four different providers on the basis of ‘gold standard' data sets obtained by manual validation and correction of cell lineages. It scores best in all three tested categories: nucleus centre detection, linkage and mitosis detection. Thus, the combined BioEmergences/Mov-IT platform can contribute to the definition of standard procedures for the reconstruction of lineage trees from 4D *in vivo* data. The validation, annotation and analysis tools provided here support detailed, large-scale cell clonal analysis and characterization of cell behaviour along the lineage tree. This leads the way to the creation of benchmarks for a new type of interdisciplinary and quantitative integrative biology.

## Results

### Overview of the BioEmergences workflow

**Figure 1:**
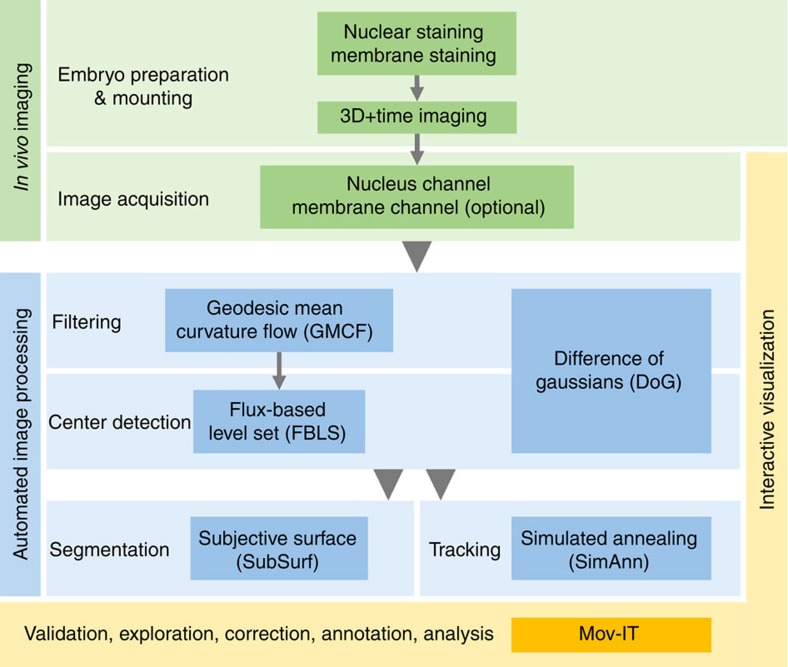
The BioEmergences reconstruction workflow. From top to bottom: successive steps starting from embryo preparation and leading to the reconstructed data, all readily available for display and analysis by the interactive visualization tool Mov-IT. Each processing step is described in greater detail in the Methods. We propose two alternative nuclear centre detection methods. Either output can be used for shape segmentation and/or cell tracking.

**Figure 2:**
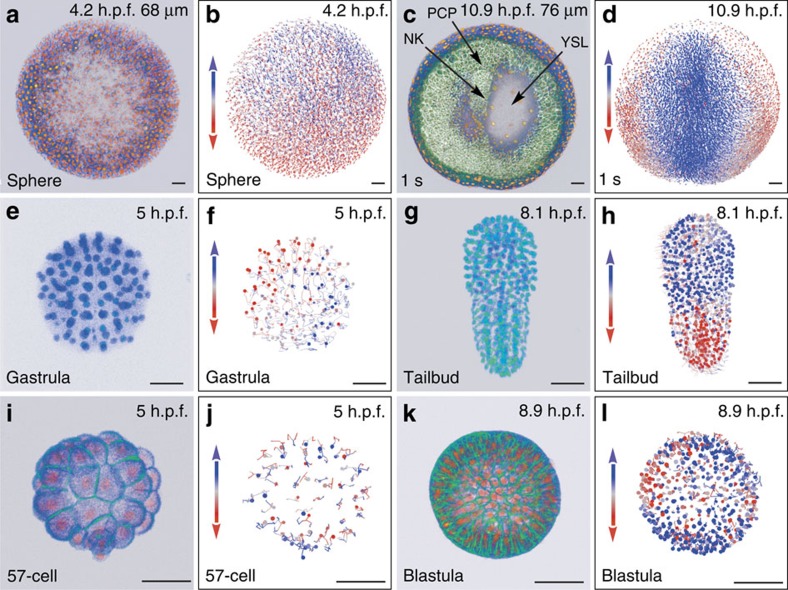
Reconstructing early embryogenesis from time-lapse optical sectioning. (**a**–**d**) *Danio rerio* data set Dr1, animal pole (AP) view; (**a**,**b**) sphere stage, volume cut at 68 μm of the AP to visualize deep cells; (**c**,**d**) 1-somite stage (1 s), volume cut at 76 μm of the AP; NK, neural keel; PCP, prechordal plate; YSL, yolk syncytial layer. (**e**–**h**) *Phallusia mammillata* data set Pm1, vegetal view, circum notochord side up; (**e**,**f**) gastrula stage; (**g**,**h**) tailbud stage. (**i**–**l**) *Paracentrotus lividus* data set Pl1, lateral view, AP up; (**i**,**j**) 57-cell stage; (**k**,**l**) blastula stage. (**a**,**c**,**e**,**g**,**i**,**k**) 3D raw data visualization (with Amira software), hours post fertilization (h.p.f.) indicated top right, developmental stage indicated bottom left, scale bars, 50 μm. (**a**,**c**,**i**,**k**) Nuclear staining from H2B-mCherry mRNA injection (in orange), membrane staining from eGFP-HRAS mRNA injection (in green). (**e**,**g**) Nuclear staining from H2B-eGFP mRNA injection (in blue-green). (**b**,**d**,**f**,**h**,**j**,**l**) Reconstructed embryo visualized with the Mov-IT tool, corresponding to (**a**,**c**,**e**,**g**,**i**,**k**), respectively. Each cell is represented by a dot with a vector showing its path over the next time steps (15 for the zebrafish data, 9 for the ascidian and sea urchin data). Colour code indicates cell displacement orientation.

The phenomenological reconstruction of embryonic cell lineage starts from multimodal 4D data, typically comprising at least one channel for the fluorescent signals emitted by cell nuclei, and possibly another one for cell membranes. Although nuclear staining is essential for cell tracking, membrane staining is necessary to assess cell morphology and neighbourhood topology. We deliver the first public version of the standalone BioEmergences workflow, with graphical user interface, able to launch a succession of algorithmic steps on two-channel raw data (Fig. 1 and Methods). Image filtering, nucleus centre detection, membrane shape segmentation and cell tracking methods were all designed and tuned to deal with the inherent noise and incompleteness of 4D imaging data generated by MLSM (Fig. 2). The BioEmergences standalone pipeline produces reconstructed data that can be directly displayed by the interactive visualization software, Mov-IT (Methods). By superimposing reconstructed data on raw data, Mov-IT adds visual inferences to create an ‘augmented phenomenology'. This allows the user to control data quality, measure the error rate, easily correct cell detection and tracking errors, and investigate the clonal history of cells and their behaviour.

The automated tracking of cells from 4D image data sets across whole living embryos involves a difficult trade-off between interdependent variables: signal-to-noise ratio, spatial and temporal resolution, imaging depth and cell survival. Microscopy techniques for *in vivo* and *in toto* imaging of developing organisms are evolving rapidly. In particular, the combination of two-photon excitation fluorescence for deep-tissue imaging over extended periods of time^20^,^21^ with parallelized microscopy based on SPIM/DSLM is a promising approach^22^, although it is not yet widely available for routine time-lapse imaging in developmental biology.

We explored here the coupling of two femtosecond lasers with two upright laser-scanning microscopes (Supplementary Fig. 1). Our methods are illustrated on 6-h spatiotemporal sequences covering the cleavage and gastrulation periods in *Danio rerio* (Dr1 and Dr2) and *Phallusia mammillata* (Pm1 and Pm2), and the cleavage stages in *Paracentrotus lividus* (Pl1 and Pl2; Fig. 2 and Supplementary Movies 1, 2 and 3). The ascidian and echinoderm embryos were imaged *in toto*, allowing the reconstruction of the complete cell lineage across entire specimens. The subvolume imaged in the developing zebrafish contained up to 9,500 cells, adding the difficulties of high nucleus density and noise increase along the imaging depth. The user can choose between a standalone version, easily deployed on a personal computer but limited in terms of scalability, and a web service with user support, made to rapidly process large data sets through parallel implementation on our computer clusters and the EGI (Supplementary Note 1 on computational speed). The latter is especially powerful for optimizing the choice of algorithm parameters under expert supervision. The comparison of the BioEmergences workflow with other available strategies demonstrates the performance and usefulness of our tools.

### Image processing steps

The BioEmergences automated image processing workflow (Fig. 1) is delivered as a standalone code that can be executed from the command line and, in part, from a graphical user interface. It starts by filtering the images and detecting the cell centres from local maxima, then performs a tracking of the cells' trajectories, with optional segmentation of the shapes of nuclei and membranes, producing in the end a digital specimen in output (Methods, Supplementary Movies 4, 5 and 6).

**Figure 3:**
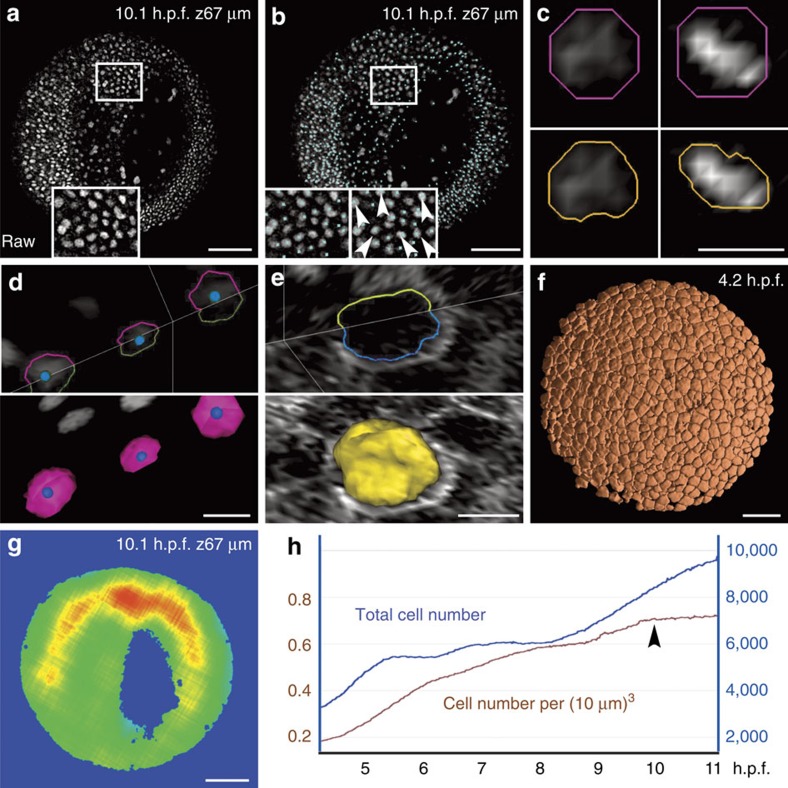
Chain of PDE-based algorithms for the 3D segmentation of embryonic cells. Results from the zebrafish data set Dr1. Scale bars in μm (100 in **a**,**b**,**f**,**g**, 10 in **c**,**d**,**e**). (**a**) Raw data image; single *z* section at 67 μm depth (orientation as in Fig. 2d), magnification in inset. (**b**) Nucleus centre detection by the FBLS method, after filtering by GMCF; approximate centres (cyan cubes) superimposed on raw data (grey levels, same orthoslice as **a**). Magnification in insets: left inset at depth 67 μm; right inset located one section deeper, showing that several centres not displayed at 67 μm were in fact correctly detected and visible below the chosen plane (white arrowheads); remaining centres can be found on other planes using the Mov-IT interactive visualization tool. (**c**) Nucleus segmentation by SubSurf method; left panels: an interphase nucleus; right panels: a metaphase nucleus; top panels: initial segmentation (pink contour) superimposed on raw data (grey levels); bottom panels: final segmentation (orange contours) superimposed on the same raw data. (**d**) Nucleus segmentation by SubSurf method; top panel: three nuclear contours superimposed on two raw-data orthoslices; bottom panel: 3D rendering of the segmented nuclei on a single orthoslice. (**e**) Membrane segmentation by SubSurf method; top panel: one cell membrane contour superimposed on two-raw data orthoslices; bottom panel: 3D rendering of the segmented membrane shape on a single orthoslice. (**f**) 3D rendering of segmented cell shapes. (**g**) Embryo shape segmentation; local cell density increasing from blue to red (same orthoslice as **a**). (**h**) Total cell number (blue curve) and cell density (brown curve) as a function of time; arrow indicates the end of gastrulation correlating with a plateau in cell density.

Filtering and nucleus centre detection algorithms based on multiscale image analysis^23^ and partial differential equations (PDEs) are particularly useful for data sets that contain a high density of nuclei, as observed in the zebrafish by the end of gastrulation and beyond. Starting from raw images (Fig. 3a), an initial image filtering step removes noise through geodesic mean curvature flow (GMCF)^24^ relying on a nonlinear geometrical PDE that can simultaneously smooth and sharpen the image^25^. Next, the position of each nucleus is found by a flux-based level set (FBLS) centre detection method (Fig. 3b)^26^. It identifies objects with ‘humps' in the image intensity function, and places nuclear centres at local maxima. This is done through a nonlinear advection-diffusion equation that moves each level set of image intensity by a constant normal velocity and curvature.

Alternatively, we also provide a module performing well with a low density of nuclei and highly contrasted images. Based on a difference of Gaussians (DoG) convolution filter, it is able to simultaneously smooth the image and keep the most salient features. In the web service implementation of the workflow, parameters of the Gaussians are interactively selected upon visual inspection of the resulting centres.

In an optional step, both nucleus and membrane geometries are automatically found by shape segmentation (Fig. 3c–e) using the subjective surface (SubSurf) technique initialized with the previously detected cell centres^27^,^28^,^29^. The numerical discretization of GMCF, FBLS and SubSurf is based on the co-volume method^30^ and its parallel implementation^19^. The SubSurf method was also used to segment the global volume of imaged embryonic tissue, useful to estimate the average cell density and its evolution through time (Fig. 3f–h). Finally, our original cell tracking algorithm inputs the list of approximate centres of cell nuclei and outputs their lineage in space and time following a three-step strategy. The first step initializes the lineage links by a nearest-neighbour heuristic method. The second step uses simulated annealing^31^, a variant of the Metropolis algorithm, to progressively enforce a set of constraints reflecting *a priori* biological knowledge. This is achieved by repeated random trials of link modification, and validation of possible changes according to a cost function, based on a weighted sum of contributing terms and a ‘temperature' parameter. The last step uses simulated annealing to link childless and motherless cells to the tree in an acceptable way, leaving open the possibility to make a posteriori changes in the detected nuclei.

### Visualization and validation

After processing, each step of the reconstruction can be interactively visualized and analysed with the Mov-IT software. Developed to address the needs of biologists investigating the potential of *in silico* embryology based on 3D+time microscopy imaging, Mov-IT allows easy validation and correction of nucleus detection and cell lineage by superimposing reconstructed data on raw data. To this aim, it provides a complete set of tools including 3D volume rendering, 2D orthoslice views, cell lineage and segmentation display.

**Figure 4:**
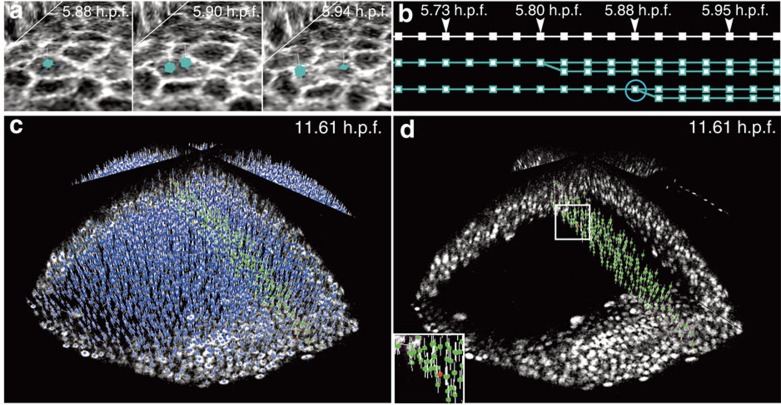
Visualization and validation of the lineage tree reconstruction (zebrafish dataset Dr1). Results from the zebrafish data set Dr1. All screenshots taken from the Mov-IT visualization interface, then tagged. (**a**) Cell division illustrated by three snapshots; time in hours post fertilization (h.p.f.) indicated top right; cell centres (cyan cubes) and cell paths (cyan lines) superimposed on two raw-data orthoslices showing the membranes (grey levels). (**b**) Flat representation of the cell lineage tree for three cell clones over 17 consecutive time steps: each cell is represented by a series of cyan squares, linked according to the cell's clonal history; the cell dividing in Fig. 4a is circled. (**c**,**d**) Nucleus centre detection in a subpopulation of cells chosen at 11.61 h.p.f. Mov-IT visualization in 'checking mode' adding short vertical white lines to the detected centres, and displaying correct nuclei in green and false positives in red; (**c**) all detected nuclei; (**d**) validated nuclei only.

The Mov-IT software was used to validate the reconstruction of our six specimens' cell lineage trees. The sea urchin (Pl1,2) and ascidian (Pm1,2) data sets were extensively checked and curated, producing quasi error-free digital specimens (gold standards) over an average of 30,000 temporal links. The fish data sets (Dr1,2) were only partly checked because of their large number of cells. More than 80,000 tracking links and 1,200 cell divisions were validated and, when necessary, corrected in the data set Dr1 chosen to establish our standard validation protocol (Fig. 4a–d, Methods, Supplementary Movies 7, 8 and 9).

The performance of our automated workflow on data set Dr1 was evaluated on three processing outputs: nuclear centre detection, linkage (by tracking cells one time step in the past) and mitosis detection. For each output, three success or error percentages were calculated: a sensitivity (representing the rate of true positives (TPs)), a false detection rate (representing false positives (FPs)) and a false negative (FN) rate. Finally, the product of centre detection sensitivity and linkage sensitivity produced a global lineage score (Methods, Supplementary Table 1 and Supplementary Fig. 2). On average over four windows of 21 time steps distributed in the first 360 time steps of data set Dr1 (1h30 out of 6h40 of imaging from prior gastrulation to the 1 somite stage), the average linkage sensitivity of BioEmergences was 97.89%, meaning that only 2.11% of the cells were missed or not correctly tracked between time steps. FP nuclei were a negligible source of tracking errors (0.21%). False trajectories, in which tracking jumped from one cell to another between two time steps were observed in a very small number of cases, too (1.03%). The performance on small organisms, sea urchin and ascidian, was close to the gold standards for the best part of the images. Cell detection and tracking were occasionally poorer, depending on the image quality, essentially the signal-to-noise ratio, which degraded with imaging depth.

**Figure 5:**
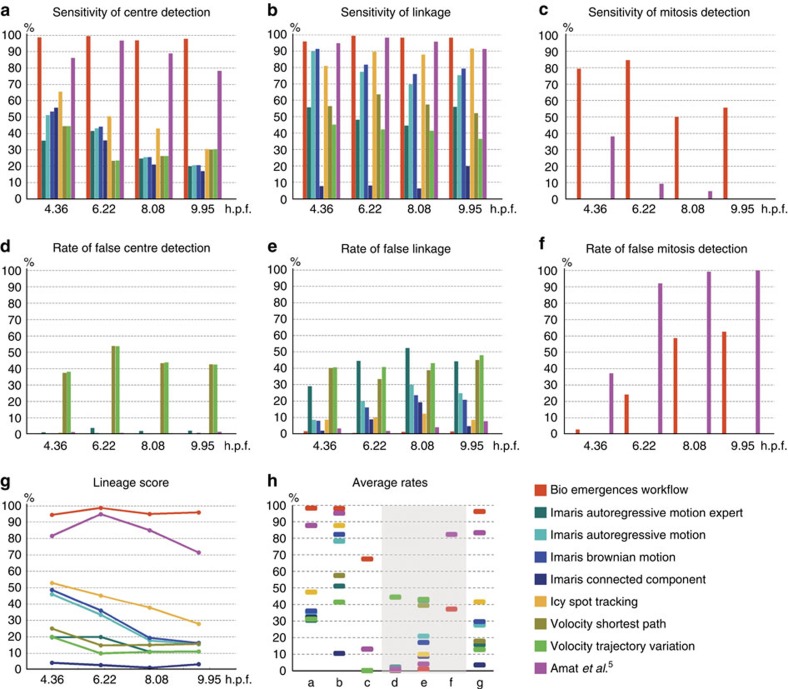
Comparative performance of nine software tools on zebrafish data set Dr1. The BioEmergences workflow presented here (red) was evaluated alongside Imaris (four shades of blue), Icy (yellow), Volocity (two shades of green) and Amat *et al*.^5^ (pink). Measurements were made by comparing the outcome of three reconstruction methods: nucleus detection, cell tracking (linkage) and mitosis detection, to a set of validated events (positions, trajectories, divisions) registered in a ‘gold standard'. This was done inside four time intervals centred in 4.36, 6.22, 8.08 and 9.95 h.p.f. (**a**) Sensitivity of nuclear centre detection, representing the rate of true positive (TP) centres. (**b**) Sensitivity of linkage, representing the rate of TP links (restricted to the subset of TP centers that possessed a validated link). (**c**) Sensitivity of mitosis detection, representing the rate of TP divisions (restricted to the subset of TP centres). (**d**–**f**) Rates of false positive (FP) centres, links and divisions (only two software tools applicable to the latter). (**g**) Global lineage score, equal to the linkage sensitivity times the centre detection sensitivity: all methods except BioEmergences deteriorate noticeably at later developmental stages. (**h**) Average rates calculated over the four time intervals: each column displays the mean heights of one of the previous seven charts. BioEmergences obtained the best results in every category: highest values in **a**–**c**,**g** (success rates), lowest values in **d**–**f** (failure rates). Formulas can be seen in Methods, detailed values in Supplementary Table 1.

The relevance and usefulness of the BioEmergences reconstruction workflow are demonstrated by comparing its outputs with those obtained on the same dataset Dr1 using state-of-the-art commercial and open-source software. Eight image processing tools from four different providers were deemed suitable to handle gigabytes of time-lapse data. Their performance was tested on the detection of nuclear centres, links and cell divisions in several time intervals (Fig. 5, Methods and Supplementary Table 1). Based on measures of TP rates, called ‘sensitivity', and FP rates, our methods produced the best results in every category. In particular, BioEmergences had the lowest rate of false linkage at 1% (Fig. 5h, column e). It also obtained an average mitosis sensitivity of 67% against 13% for Amat *et al*. and 0% for the rest (Fig. 5h, column c), meaning that the other tools were actually not designed to join lineage branches through the detection of divisions. Our software and Amat *et al*.'s software were the only ones allowing the linkage of one mother cell at time step *t* to two daughter cells at time step *t*+1. In essence, mitosis detection is a special task that only two packages among the nine that we tested were able to solve. Typically, it is at the time of anaphase and telophase that an algorithm can detect two separate nuclei, and as soon as it does it must also link them back to their mother. If this is not accomplished, the lineage tree will lack a junction. Even when a mitotic event is missed, most methods are still able to correctly track the resulting two cells, but these cells will not be properly annotated as daughters (Supplementary Movie 10).

Overall, our workflow reached an average ‘lineage score' of 96% (the product of centre sensitivity and linkage sensitivity), whereas Amat *et al*. had 83% and all other tools remained below 50% (Fig. 5h, column g). Behind these average values, there was also a noticeable degradation of performance at later developmental stages in all systems except BioEmergences (Fig. 5g).

### Analysis of cell fate and proliferation in digital specimens

**Figure 6:**
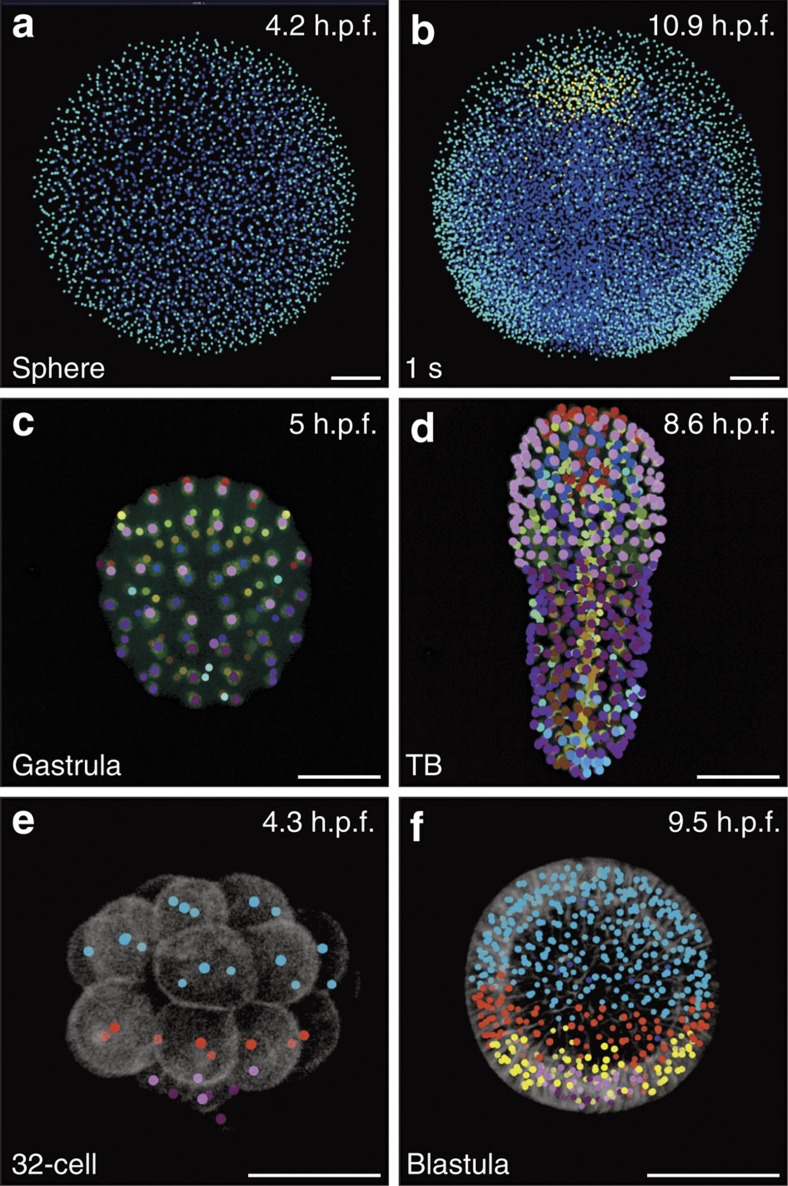
Automated fate map propagation. Cells were manually selected according to their identity or fate, using the interactive visualization tool Mov-IT. Developmental stages indicated bottom left, developmental times in h.p.f., top right. All scale bars, 50 μm. (**a**,**b**) Three cell populations in *Danio rerio* specimen Dr1, animal pole view; (**a**) sphere stage, ventral (anterior) up, enveloping layer cells in cyan, epiblast cells in blue; (**b**) 1-somite stage, same colour code plus hypoblast cells in yellow. (**c**,**d**) Fate map in *Phallusia mammillata* specimen Pm1, vegetal view, circum notochord up, colour code as in ref. 33; (**c**) gastrula stage; (**d**) tailbud (TB) stage with automated propagation along the cell lineage of the fate map shown in **c**. (**e**,**f**) Cell populations in *Paracentrotus lividus* specimen Pl1, lateral view, animal pole up; small micromeres in dark purple, large micromeres in light purple; mesomeres in cyan. (**e**) 32-cell stage, macromeres in red; (**f**) blastula stage, Veg1 cell population in red, Veg2 cell population in yellow.

The cell lineage tree is the cornerstone of a detailed understanding of morphogenetic processes (Supplementary Note 2 on *in silico* fate mapping). Digital specimens, such as the examples presented here, constitute a unique source of in-depth knowledge into embryonic patterning and the individuation of morphogenetic fields (Fig. 6). In the zebrafish embryo, cells from the epiblast, hypoblast or epithelial enveloping layer were distinguished according to their position and behaviour^32^, and selected in the digital specimen with the Mov-IT tool. The selected cell populations were tracked, either backward or forward, revealing their relative movements with resolution at the cellular level during gastrulation stages (Fig. 6a,b and Supplementary Movie 11). The more stereotyped cell lineage of the tunicate *Phallusia mammillata* allowed us to transpose the state-of-the-art fate map established at the 110-cell stage^33^. Cells were marked in the digital specimen according to their presumptive fate (Fig. 6c,d and Supplementary Movie 12). The propagation of the 110-cell stage fate map along the cell lineage revealed the patterning of all the morphogenetic fields with a temporal resolution in the minute range. In the sea urchin embryo at cleavage stages, the cell membrane channel was used to mark cells according to their volume. This led to the identification of the three cell populations: micromeres, macromeres and mesomeres, organized along the vegetal-animal axis, with further segregation of macromeres into Vg1 and Vg2 subtypes, and micromeres into small and large subtypes^34^ (Fig. 6e,f and Supplementary Movie 13). The propagation of colours along the cell lineage showed that, despite limited cell dispersion, the cellular organization in the sea urchin embryo varied from one embryo to another.

The Mov-IT interface was also designed for fast import of processed data files in order to perform a systematic analysis of cell and tissue properties (Mov-IT tutorial). This is illustrated by the analysis of the evolution of cell proliferation and cell density in our sea urchin, ascidian and zebrafish specimens (Supplementary Fig. 3). Further statistical analysis and measurements are expected to contribute in a feedback loop to theoretical models and numerical simulations (Supplementary Fig. 4).

## Discussion

The BioEmergences reconstruction pipeline, accessible through a simple graphical user interface, was designed to run as quickly and efficiently as possible from the acquisition of microscopy images to the display of cell lineage and cell segmentation aligned with raw data. Although the standalone version was crafted for convenient processing of small data sets on a laptop computer, there is no size limit on the data that can be uploaded to the BioEmergences web service through iRODS, an open-source data management software and the OpenMOLE engine^14^ for data processing on the EGI.

The concept of ‘augmented phenomenology', coined to describe the overlay of raw and reconstructed data required for validation, correction and analysis, is fully exploited using the custom-made Mov-IT software. This tool serves in particular to demonstrate that the reconstructed cell lineages meet the best quality of precision and accuracy. So far, cell lineage data can only be validated by eye inspection or by comparison with available gold standards, which are established manually and cross-checked by at least two experts. Corrected data sets are considered error-free as long as experts do not dispute this conclusion. Although gold standards themselves depend on what the eye can achieve, it is generally accepted that automated image processing software is still on average less effective than human vision. Our cross-software comparison method based on a set of validated events (cell positions, temporal links, divisions) is a step in the direction of standardized validation and comparison protocols. All the results led to the conclusion that the BioEmergences workflow achieved the best performance on standard data produced by MLSM imaging with a temporal resolution chosen to explore either a small specimen (sea urchin or tunicate embryo) or up to one-third of the zebrafish gastrula. The software provided by Amat *et al*. was the next best, but its performance dropped faster than BioEmergences with the increase in cell density and tissue thickness.

The cell tracking pipeline delivered here should be useful for a large variety of model organisms that have adequate optical properties to allow the acquisition of 3D+time data sets, including a channel for stained nuclei. The requirements in terms of spatial and temporal resolution and signal-to-noise ratio are easily fulfilled with commercial microscopy setups, either MLSM or SPIM. However, the accuracy of cell lineage reconstruction depends to a great extent on the quality of the data. Biologists who try for the first time the automated processing of their time-lapse images might have to adjust their staining and imaging scheme to increase the quantity of information collected, especially across tissue depth. Image processing outcome also depends on the selection of algorithm parameters. This might require a few trials in the standalone version, if the default parameters do not already lead to satisfactory results. The web service version, on the other hand, offers the advantage of fast processing on a grid infrastructure, allowing the concurrent execution of hundreds of parameter combinations that can be easily explored by the user through Mov-IT, as done for the DoG centre detection method.

The detection of cell nuclei is a critical step, as it is used not only for cell tracking but also for nucleus and membrane shape segmentation, which must be performed on validated nuclei to avoid major errors but is computationally expensive. Although the standalone software performs well on a few selected cells, full-scale shape segmentation can be achieved in a reasonable amount of time only on a computing cluster such as the one accessible through the BioEmergences web service. Segmentation output can also be verified with Mov-IT, but its quantitative validation remains an issue^35^.

The public availability of the BioEmergences platform and its application to the embryogenesis of model organisms is intended to open the path to *in silico* embryology based on digital specimens. We illustrate this ambition with fate-map studies. The possibility of constructing complete fate maps in digital specimens, as we achieved for the tunicate embryos, is a revolution in the field. Finally, although delivering here a standalone software, we also expect to initiate through the web service option a synergistic effort of the scientific community towards further validation, correction and annotation of digital specimens.

## Methods

### Embryo staining and mounting

Wild-type *Danio rerio* (zebrafish) embryos were injected at the one-cell stage with 200 pg H2B-mCherry and 200 pg enhanced green fluorescent protein (eGFP)-HRAS mRNA prepared from PCS2+ constructs^36^,^37^. Although mCherry was not as bright as eGFP and bleached more through imaging, this colour combination was the best compromise for a proper staining of cell membranes and further segmentation. Injected embryos were raised at 28.5 °C for the next 3 h. Embryos were mounted in a 3-cm Petri dish with a glass coverslip bottom, sealing a hole of 0.5 mm at the dish centre, where a Teflon tore (ALPHAnov) with a hole of 780 μm received the dechorionated embryo. The embryo was maintained and properly oriented by infiltrating around it 0.5% low-melting-point agarose (Sigma) in embryo medium^38^. Temperature control in the room resulted in ∼26 °C under the objective, slightly slowing down development with respect to the standard 28.5 °C developmental table^32^ (Supplementary Table 2). After the imaging procedure, the embryo morphology was checked under the dissecting binocular and the animal was raised for at least 24 h to assess morphological defects. Embryo survival depended on total imaging duration, average laser power and image acquisition frequency (time step Δ*t*). The zebrafish data sets Dr1 and Dr2 were imaged through a standardized procedure with Δ*t*<150 s allowing up to 120 sections per time point with an average laser power of 80 mW delivered to the sample for more than 10 h without detectable photodamage. Lowering the laser power to less than 60 mW and increasing Δ*t* up to 3.5 min allowed imaging embryos for more than 20 h, then raising them to adulthood. These conditions were used to obtain up to 320 sections at a 400 Hz line scan rate (bidirectional scanning) or 200 Hz to improve signal-to-noise ratio.

Oocytes from *Phallusia mammillata* (ascidian) were dechorionated and injected as described^39^ with 1 mg ml^−1^ H2B-eGFP mRNA prior fertilization. Membrane staining was obtained through continuous bathing in artificial sea-water containing FM 4–64 (Life Technologies) at a concentration of 1.6 μg ml^−1^. Embryos were deposited in a hole made in 1% agarose in filtered sea water at the centre of a 3-cm Petri dish.

Oocytes from *Paracentrotus lividus* (sea urchin) were prepared and injected as described^40^ with 150 μg ml^−1^ H2B-mCherry and 150 μg ml^−1^ eGFP-HRAS synthetic mRNA. Embryos were either maintained between slide and coverslip covered with protamin^41^ or embedded in 0.25% low-melting-point agarose sea water at the centre of a 3-cm Petri dish.

### Image acquisition

Imaging was performed with Leica DM5000 and DM6000 upright microscopes SP5 MLSM, equipped with an Olympus 20/0.95NA W dipping lens objective or a Leica 20/1NA W dipping lens objective. Axial resolution at the sample surface (1.5 μm) was estimated by recording 3D images of 0.1 or 1 μm fluorescent polystyrene beads (Invitrogen) embedded in 1% agarose. For the zebrafish specimen Dr1, the field size was 700 × 700 μm^2^ in *x*, *y* and 142 μm in *z*, with a voxel size of 1.37 × 1.37 × 1.37 μm^3^ and a time step of 67 s. For the zebrafish Dr2, the field size was 775 × 775 μm^2^ in *x*, *y* and 164 μm in *z*, a cubic voxel of edge 1.51 μm and a time step 153 s. For the ascidian Pm1, these dimensions were 384 × 384 μm^2^ in *x*, *y*, 165 μm in *z*, voxel 0.75 × 0.75 × 1.5 μm^3^ and time step 180 s. For the ascidian Pm2: 353 × 353 μm^2^ in *x*, *y*, 188 μm in *z*, voxel 0.69 × 0.69 × 1.39 μm^3^ and time step 180 s. For the sea urchin Pl1: 280 × 280 μm^2^ in *x*, *y*, 86 μm in *z*, voxel 0.55 × 0.55 × 1.09 μm^3^ and time step 207 s. For the sea urchin Pl2: 266 × 266 μm^2^ in *x*, *y*, 82 μm in *z*, voxel 0.52 × 0.52 × 1.05 μm^3^ and time step 180 s. For two-colour acquisition, simultaneous two-photon excitation^42^ at two different wavelengths (1,030 and 980 nm) was performed with pulsed laser beams (T-pulse 20, Amplitude Systèmes and Ti-Sapphire femtosecond oscillator Mai Tai HP, Newport Spectra physics, respectively). Details of the optical bench are provided in Supplementary Fig. 1. Raw-data movies were made with the Amira software (Mercury Computer Systems).

### Image processing algorithms

Explanation of the parameters and their useful range, along with the specific values used to process data sets Dr1-2, Pm1-2 and Pl1-2, are all provided in Supplementary Table 3. Filtering by GMCF relied on the following PDE^25^,^43^,^44^:


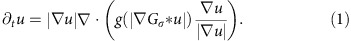


It was accompanied by the initial condition 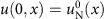, or 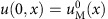, where 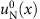 and 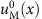 are the image intensities of the nuclei and the membranes, respectively, depending on which channel was filtered. In the GMCF model, the mean curvature motion of the level sets of image intensity is influenced by the edge indicator function *g*(*s*)=1/(1+*Ks*^2^), *K*⩾0, applied to the image intensity gradient pre-smoothed by convolution with a Gaussian kernel *G*_*σ*_ of small variance *σ*. Details of the numerical method for solving equation (1) and its (parallel) computer implementation are given in refs 25, 43.

Centre detection by FBLS defined the nuclei as the local maxima of a smoothed version of the original image. Our algorithm was based on the following PDE^26^:


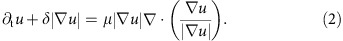


where the initial condition was given by the intensity function of the filtered nucleus image 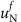. Equation (2) represents the level-set formulation for the motion of isosurfaces of solution *u* by a normal velocity *V*=*δ*+*μk*, where *δ* and *μ* are constants and *k* is the mean curvature. Owing to the shrinking and smoothing of all level sets, the function 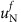 was simplified, and we observed a decrease in the number of spatial positions of local maxima, which could be used as approximate nuclear centre positions. Details of the numerical solution to equation (2) can be found in refs 26, 43.

Alternatively, smoothing and centre detection could be achieved by DoG, a convolution of the image with two Gaussians of different standard deviations, here 1.5–2.5 μm and 12–16 μm respectively. Their difference was calculated and the gray values above a threshold between 1 and 10% were selected. This allowed to simultaneously smooth the image and keep the most significant objects. We ran multiple simulations combining different possible values of standard deviations and thresholds. Optimal values were visually chosen with Mov-IT by interactively checking the detection results.

Nucleus and membrane segmentation extracted the shapes of cell nuclei and/or membranes by evolving an initial segmentation function based on the subjective surface (SubSurf) equation^27^:


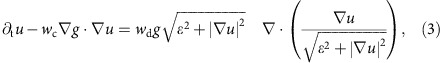


where 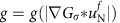 for nucleus shapes, 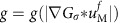 for membrane shapes, and *ɛ*, *w*_c_, *w*_d_ are parameters. A detailed description of the role of these parameters, with explicit and semi-implicit numerical schemes, along with a discussion of the computational results is given in refs 28, 29. The same equation (3) is also used for the overall embryo shape segmentation^29^.

Our cell tracking algorithm uses simulated annealing (SimAnn). It takes in input the list of approximate centres of cell nuclei detected at each time step, and produces in output a lineage ‘forest', a graph equal to the union of several disjoint trees, in which each cell present at the first time step was the root of a lineage tree. A graph is composed of a set of edges, or ‘links', each of them connecting a nucleus centre at time *t* to a nucleus centre at time *t*+1. The algorithm was implemented by an automated three-stage process:

First, edges between centres at consecutive time steps were initialized using a nearest-neighbour heuristic method. This created a set of links that were not necessarily biologically plausible.

Second, simulated annealing^31^, a variant of the Metropolis algorithm^45^, was used to progressively enforce a set of predefined constraints summarizing together a certain number of biological requirements on the lineage forest, most notably: each cell beyond the first time step should come from a single cell at a previous time step; each cell should have a single ‘mother' (a corresponding centre at *t*−1); no cell should have more than two ‘daughters' (corresponding centres at *t*+1); no cell should disappear (there is no cell death at these developmental stages in the chosen species—an assumption invalid in other cases, such as mammalian preimplantation embryos); divisions should not occur too frequently; cell displacements should be bounded and so on. At the start, a finite set of allowed link modifications and a cost function measuring the departure from the constraints were defined. Then, the algorithm relied on random link visits, and acceptance or rejection of a potential link change based on its cost. More precisely, was the weighted sum of local contributions, each addressing one of the *a priori* biological requirements. The cost function weights were selected after some initial trials of the algorithm and visual inspection of the resulting lineages, then recorded in a configuration file and kept constant during computation. This second stage consisted of repeatedly selecting and tentatively modifying a link at random from the list of permitted moves, then evaluating the resulting change through the cost function. In case of cost decrease, the candidate change was systematically accepted, whereas in case of cost increase, it was accepted with a probability proportional to exp(−Δ/*T*), where *T* is a ‘temperature' parameter that was progressively lowered (‘annealed') over time so that fewer and fewer breaches to the constraints were accepted. The temperature decrease schedule was linear with time. The cost function also included terms to penalize deformations, to favour symmetry in the behaviour of sister cells, to add noise-countering inertia, to bound speed, and to account for division times.

Third, the final goal was to minimize false-positive and false-negative errors in the nuclear detection steps. This was achieved by looking at the whole biological coherence of the lineage tree and identifying lineage gaps or cells that lived only a few time steps. The algorithm could then delete the centres of short branches or introduce ‘virtual centres' to regain continuity. To find a best solution, it used simulated annealing again. A parallel implementation was written, which partitioned space-time into different ‘cylinders' that were run on different processors, then merged the results.

### Validation protocol

Obtaining a complete ‘gold standard' annotated reference, even for small animals such as the ascidian and sea urchin embryos, was possible only with considerable human effort. The *Phallusia* and *Paracentrotus* data sets, Pm1-2 and Pl1-2, were almost completely checked and curated. Cell tracking accuracy depended on the imaging depth, along which the signal-to-noise ratio degraded. Owing to the decrease in image quality with depth, there remained a few errors for which there was no solution, even through manual expertise. We curated around 25,000 temporal links in Pl1, 22,000 in Pl2, 30,000 in Pm1 and 40,000 in Pm2. All divisions were also fully annotated. The four digital embryos produced were validated by two independent experts.

In the case of the zebrafish, however, a complete gold standard was almost impossible due to a number of cell samples of the order of several million. Our gold standard for the *Danio* data set Dr1 was obtained by a large-scale curation strategy: (i) all detected nuclear centres were completely annotated inside 54 spatial boxes of average size 340 × 70 × 140 μm^3^ in *x*, *y*, *z*, over ten time steps starting at various sample times *t*_0_∈{1, 20, 100, 200, 300, 350}. By this method, 25,123 links were manually checked, comprising 3,262 links for *t*_0_=1 (from *t*=1 to *t*=10), 2,735 for *t*_0_=20 (from *t*=20 to *t*=29), 3,005 for *t*_0_=100, 8,736 for *t*_0_=200, 5,474 for *t*_0_=300 and 1,911 for *t*_0_=350. This strategy insured even sampling throughout the image data both in time and space (Fig. 4). (ii) As part of the investigation of the zebrafish fate map, several cell clones were manually validated and when required corrected along with the whole data set, corresponding to 50,503 additional curated temporal links and the corresponding curated nuclei. (iii) An entire layer of epithelial cells was completely curated at one specific time, *t*=4. (iv) In addition, a large number of links were randomly checked, bringing the total to 80,428. (v) Finally, more than 600 mitoses were checked.

### Accuracy estimation protocol

Taking the consistent and representative gold standard of data set Dr1 as a basis, we were able to automatically identify and count various error types in the reconstructed embryos (Supplementary Table 1). This produced TP, FP and FN numbers, which were used as a performance metric for BioEmergences and other software tools. The accuracy of a given processing workflow was evaluated on three outputs: nuclear centre detection, linkage (tracking cells one time step in the past) and mitosis detection.

By definition, the sum of TP and FN centres was equal to the total number of centres in the gold standard. This relationship did not hold, however, for TP and FN links or divisions because counting those events was restricted to the subset of TP centres. For links, FN was the sum of the numbers of ‘wrong links' (WL) and ‘missing links' (ML). This is because WL, which connect a cell to a wrong target, contributed both to FP (by creating new links that do not exist) and to FN (by missing the correct links), whereas ML, which correspond to a cell without any link, contributed only to FN.

Then, for each output, three types of success and error percentages were measured: a ‘sensitivity', equal to TP/(TP+FN), a ‘false detection rate', equal to FP/(TP+FP) and a ‘FN rate', equal to FN/(TP+FN). Finally, a ‘global lineage score' was calculated as the product of centre detection sensitivity and linkage sensitivity. The rationale for this formula is that linkage alone could appear successful even if many centres were missing, therefore it should be weighted by the actual proportion of detected centres.

### Software performance and comparison

We identified eight state-of-the-art tools most relevant for our benchmark comparison: Icy (Spot Tracking, http://icy.bioimageanalysis.org), Imaris (Autoregressive Motion Expert, Autoregressive Motion, Brownian Motion and Connected Component, http://www.bitplane.com/imaris/imaris), Volocity (Shortest Path and Trajectory Variation, http://cellularimaging.perkinelmer.com/downloads) and the last method published by Amat *et al*.^5^,^46^. For each software tool, if a command line mode was available, we ran several reconstructions using different sets of parameters and selected the most advantageous one. For example, in the case of Amat *et al*., we found that an optimal configuration was backgroundThreshold=16 and persistanceSegmentationTau=0. If the software provided only a visualization interface, we produced the best reconstruction by visual inspection based on a few parameter variations.

We used data set Dr1 in input to all methods, preprocessed through our filtering algorithm (GMCF, 5 iterations). The nucleus detection and tracking outputs were compared with our gold standard data. To assess nucleus detection, we explored the neighbourhood of validated centres by looking for other detected centres at a distance of 0.2–0.6 times the average internuclear distance. The number of correct links was estimated by inspecting the subset of detected nuclei labelled TP that also possessed a link in the gold standard, and counting the correct links from *t* to *t*−1. Mitosis detection was also assessed within the set of TP nuclei. All measurements were made over four separate windows of 21 time steps each: from *t*=0 to *t*=20 (corresponding to 4.36±0.18 h.p.f.), from *t*=100 to *t*=120 (6.22 h.p.f.), from *t*=200 to *t*=220 (8.08 h.p.f.) and from *t*=300 to *t*=320 (9.95 h.p.f.). The final sensitivity, false detection and FN rates were averaged over these four intervals. Detailed scores are shown in Supplementary Table 1.

### Data sets and software

The standalone BioEmergences workflow, the visualization tool Mov-IT and the six *in vivo* 3D+time image data sets are provided online at http://bioemergences.iscpif.fr/bioemergences/openworkflow-datasets.php. For each specimen, we provide the 4D raw-data images and the corresponding reconstructed embryo. The parameters used to process these data sets are provided in Supplementary Table 3.

Our BioEmergences platform is also available as a web service at http://bioemergences.iscpif.fr/workflow/, which offers users customized assistance in addition to fast processing. The online workflow architecture relies on iRODS, an open-source data management software, and the OpenMOLE engine^14^, a middleware platform facilitating the experimental exploration of complex systems models on a computing cluster, which leverages the power of the EGI. Interested users are invited to request access to these resources by sending an email to nadine.peyrieras@cnrs.fr.

## Additional information

**How to cite this article:** Faure, E. *et al*. A workflow to process 3D+time microscopy images of developing organisms and reconstruct their cell lineage. *Nat. Commun.* 7:8674 doi: 10.1038/ncomms9674 (2016).

## Supplementary Material

Supplementary Figures, Supplementary Tables, Supplementary Notes and Supplementary References. Supplementary Figures 1-4, Supplementary Tables 1-3, Supplementary Note 1-2 and Supplementary References

Supplementary Movie 1 Embryo imaged from sphere stage to 1-somite stage. Left panel: view from the animal pole inside the blastoderm. Right panel: view from the outside of the blastoderm and upper sections removed (down to 68 μm below the surface). Nuclei in blue-green, membranes in orange-red (Amira visualization software).

Supplementary Movie 2 Embryo imaged in toto from gastrulation to tailbud stage, animal view, circum notochord up. Nuclei in blue-green (Amira visualization software).

Supplementary Movie 3 Embryo imaged in toto from 32-cell stage to blastula stage, lateral view. Nuclei in blue-green, membranes in yellow-orange (Amira software visualization).

Supplementary Movie 4 Embryo reconstructed from sphere stage to 1 somite stage, first viewed from the animal pole, then from inside the blastoderm by the late shield stage. Detected nuclei are represented by cubes, and cell trajectories over the next time steps by lines. Color code from blue to red through gray indicates cell velocity from 0 to 3 μm/min (Mov-IT visualization tool).

Supplementary Movie 5 Embryo reconstructed in toto from gastrulation to tailbud stage, animal view, circum notochord up. Detected nuclei are represented by dots, and cell trajectories over the next time steps by lines. Color code from blue to red through gray indicates cell velocity from 0 to 3 μm/min (Mov-IT visualization tool).

Supplementary Movie 6 Embryo reconstructed in toto from 32-cell stage to blastula stage, lateral view. Detected nuclei are represented by cubes, and cell trajectories over the next time steps by lines. Color code from blue to red through gray indicates cell velocity from 0 to 3 μm/min (Mov-IT visualization tool).

Supplementary Movie 7 We focus during gastrulation on one cell (in green) selected from the hypoblast, and a few other cells (in pink) selected from the epiblast. Raw data is displayed as a single orthoslice (in gray levels), which is automatically adjusted to keep track of the green cell over consecutive time steps. When the selected cell divides, its progeny inherits the green label and the raw data orthoslice is automatically adjusted on one of the daughters (Mov-IT visualization tool).

Supplementary Movie 8 Selected cells including eight progenitors of the primary notochord are highlighted with colored dots at the onset of gastrulation. One cell (in yellow) is kept at a fixed position throughout the movie. Color code is propagated along the tracking to reveal the cells' clonal history and displacement in space and time. A single orthoslice of the raw data (in gray levels) is automatically adjusted to follow the selected yellow cell. The other cells do not necessarily remain in the same plane, but are accurately tracked. The whole dataset was validated and corrected to become a gold standard (Mov-IT visualization tool).

Supplementary Movie 9 A raw data orthoslice displaying the membrane channel is adjusted to follow a single micromere highlighted in green (and kept at a fixed position throughout the movie). Its clonal descendants (also in green) do not necessarily remain in the same plane, but are accurately tracked. The whole dataset was validated and corrected to become a gold standard (Mov-IT visualization tool).

Supplementary Movie 10 A single cell is selected at time step 62 (in yellow). Its lineage indicates a single branch with no division. Following its trajectory step by step shows that at time step 71, it actually divides and its color is propagated to only one of its daughters, indicating that the algorithm (SimAnn) did not detect the mitotic event. In the checking mode of the interactive visualization software Mov-IT, the yellow cell at time t70 is linked to its blue daughter at t71. Fixing this link proved being sufficient to recover the complete clonal history of the yellow cell from t62 to the end of the time lapse as it two daughters were otherwise properly tracked.

Supplementary Movie 11 Three cell populations identified according to their position and morphogenetic movements: epiblast cells (in blue), hypoblast cells (in yellow) entering the imaged volume by 5.70 hpf, enveloping layer (EVL) cells (in cyan) highlighted between 4.24 hpf and 5.66 hpf by a 3D Delaunay triangulation joining the nucleus of neighbor cells (Mov-IT visualization tool).

Supplementary Movie 12 Starting at the gastrula stage, animal view and circum notochord up, cells are labeled by a color code according to the state-of-the-art description of their fate13. The color code is automatically propagated along the cell lineage. Detected nuclei are represented by dots, and cell trajectories over the next time steps by lines (Mov-IT visualization tool).

Supplementary Movie 13 Starting at the 32-cell stage, lateral view, detected nuclei are represented by dots (size as a function of nucleus depth), and cell trajectories over the next time steps by lines. 3D rendering of the membrane raw data. Small micromeres (in dark purple), large micromeres (in light purple), mesomeres (in cyan), macromeres (in red) before the specification of Veg1 and Veg2 populations, then Veg1 cell population (in red) and Veg2 (in yellow) (Mov-IT visualization tool).
