# Supplementary material for: A workflow to process 3D+time microscopy images of developing organisms and reconstruct their cell lineage
Source: Nat Commun. 2016 Feb 25;7:8674. doi: 10.1038/ncomms9674 (PMC4773431; doi:10.1038/ncomms9674)
Supplement: Supplementary Figures, Supplementary Tables, Supplementary Notes and Supplementary References. — Supplementary Figures 1-4, Supplementary Tables 1-3, Supplementary Note 1-2 and Supplementary References [file ncomms9674-s1.pdf]

Supplementary Information

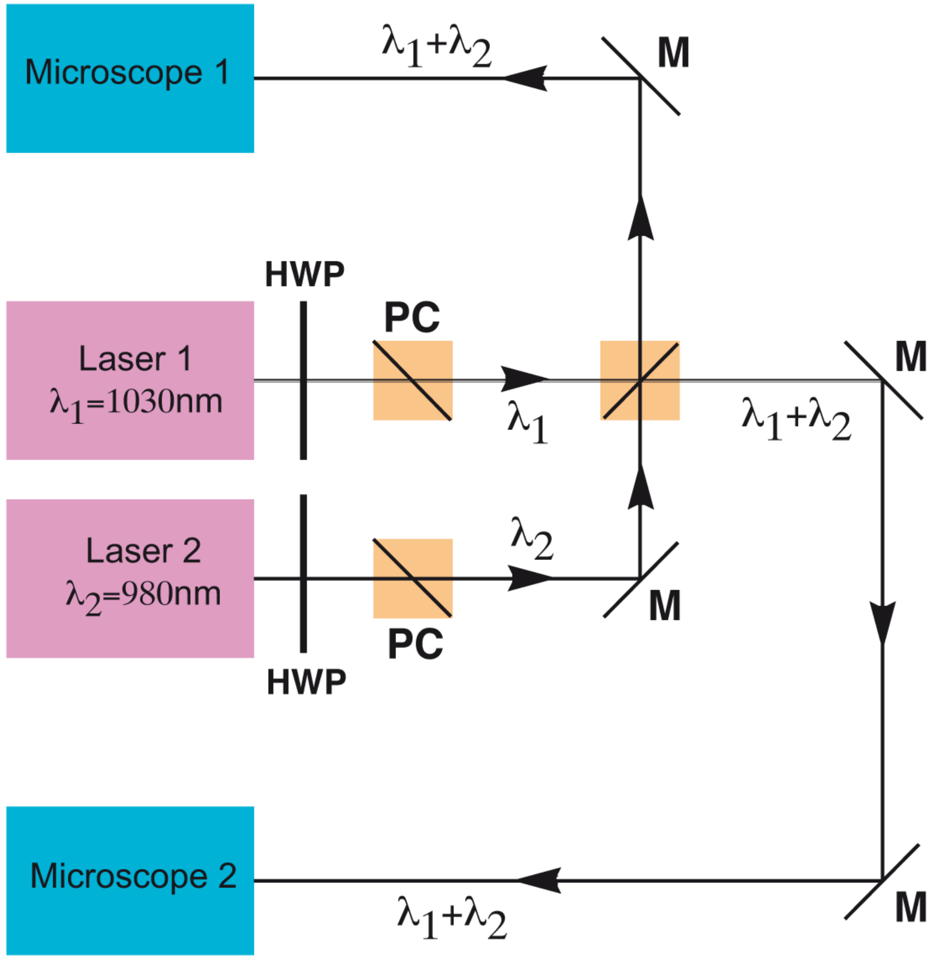

Supplementary Figure 1 | MLSM imaging setup. Laser 1: ytterbium mode-locked laser, with linear polarization, maximum power 1.1 W, wavelength λ_1 = 1030 nm, repetition rate 50 MHz, pulse duration 200 fs. Laser 2: tunable Ti-Sapphire mode-locked laser used at fixed wavelength λ_2 = 980 nm, with linear polarization, maximum power 1 W at λ_2 = 980 nm, repetition rate 80 MHz, pulse duration < 100 fs. HWP: achromatic half-wave plate. PC: polarizing cube (HWP+PC is used as a hand power controller). BS: 50/50 beamsplitter (broadband, non-polarizing cube) to transform two separate beams with distinct wavelengths into two dual-wavelength beams with equal optical power. M: broadband dielectric mirror.

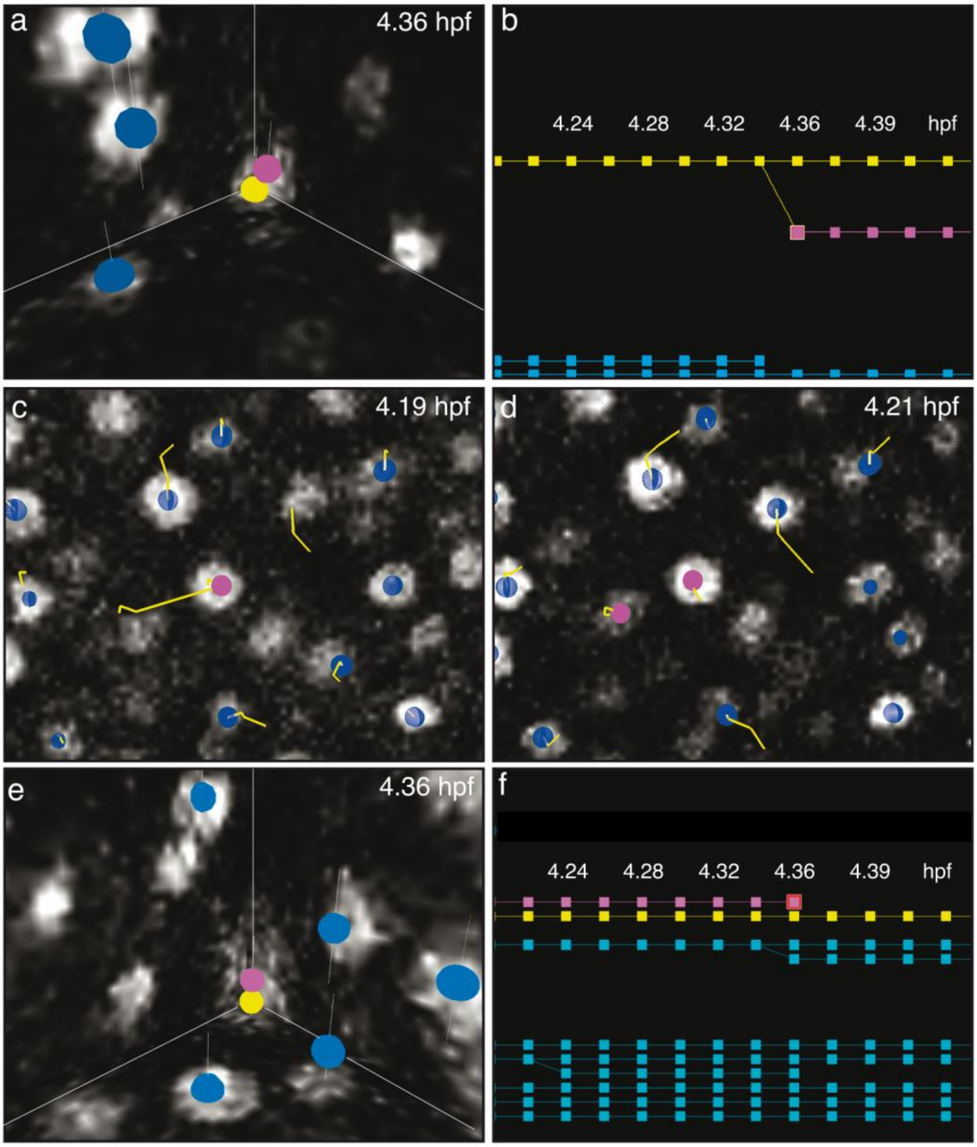

Supplementary Figure 2 | Eye inspection of false positive events. The automated comparison of the reconstructed data with the gold standard from zebrafish dataset Dr1 provided a list of false positive events that were verified with Mov-IT (Supplementary Software 2). Visual inspection demonstrated the validity of the conclusions drawn from our comparison protocol (Fig. 5 and Supplementary Table 3). Three examples of false positives in the reconstructed data obtained with Amat et al.'s software[1] are presented here. (a,b) False positive nucleus detection creating a lineage branch. (a) Two approximate centers (pink and yellow) are found inside a single real nucleus, displayed at the intersection of three raw-data orthoslices (gray levels). In "checking mode", Mov-IT extends detected centers with a short vertical white line to signal their presence across other *z* sections where they may not be visible. (b) Flat representation of the lineage tree with Mov-IT showing that this event is associated with a false positive division, since the two centers are interpreted as having the same mother cell. The false positive nucleus remained visible over the next time steps. (c,d) False positive cell division, displayed in a single raw-data orthoslice (gray levels). (c) The putative mother is labeled with a pink dot. Future trajectories over the next three time steps are indicated by thin yellow lines. (d) A neighboring cell was incorrectly picked to be a daughter cell and colored in pink, too, since Mov-IT propagates colors assigned to selected cells along their lineage. (e,f) False positive nucleus detection ending a lineage branch. (e) Same display mode as (a) and, coincidentally, at the same time. (f) Flat representation of the lineage tree with Mov-IT showing that this particular false positive event actually originated earlier, then disappeared at 4.36 hpf.

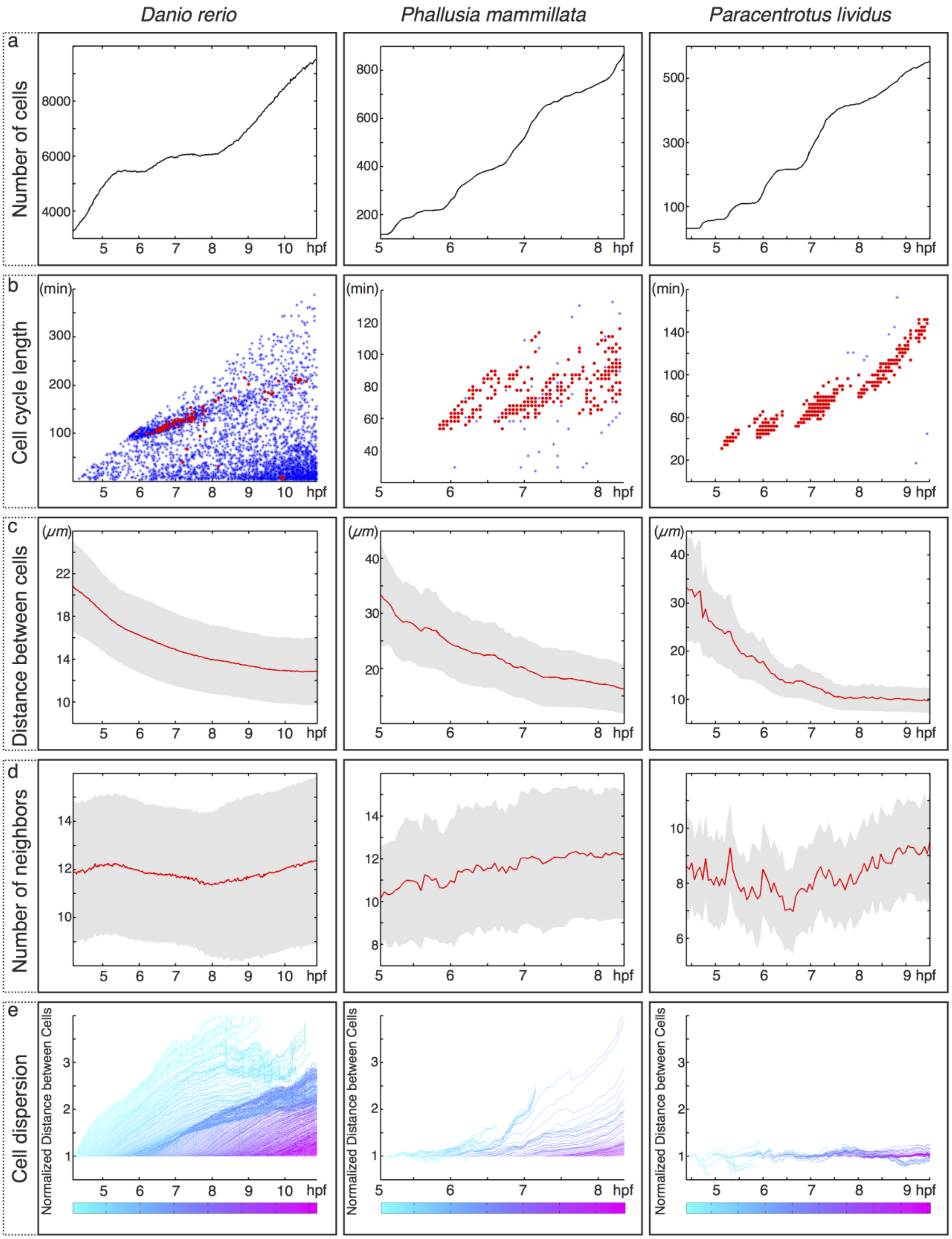

Supplementary Figure 3 | Cell proliferation rate and cell dispersion along the lineage tree. Left column: zebrafish dataset Dr1. Center column: ascidian dataset Pm1. Right column: sea urchin dataset Pl1. (a,b) The successive cell division cycles in the developing ascidian or sea urchin embryos were revealed by plotting (a) the total cell number and (b) the cell cycle length (time between two consecutive divisions) as functions of time, in hours post fertilization (hpf). In blue: all detected and tracked cells; in red: cells validated by eye inspection. Cell division synchrony was more prominent in the sea urchin. For the

zebrafish embryo, analyzed here at late blastula stages, the interpretation of the increase in cell number was highlighted by the quantification of cell density and internuclear distance (see next row). Whereas the sea urchin and ascidian reconstructed specimens were extensively corrected by experts to define gold standards, we only validated and corrected subsets of the zebrafish clones. The characteristics of the validated cells (in red) were consistent with a linear progression of cycle length for most cells throughout the blastula and gastrula stages. However, the dispersion of cycle length around the average, beside suggesting a certain number of errors in the detection of cell divisions (especially at late developmental stages), also highlighted a greater diversity of behaviors. In this respect, the ascidian *Phallusia mammillata* showed the largest variety of specific proliferation rates along the lineages. (c) Average distance between pairs of neighboring cell nuclei through divisions as a function of hpf (error margin in gray). Cells' neighborhoods are calculated by 3D Delaunay triangulation using the Computational Geometry Algorithms Library (CGAL)[2]. In all three species, this average distance decreased during early embryogenesis and converged to a minimal value around 10µm, corresponding to an average cell diameter. This observation fits with the plot of cell density, obtained by segmentation of the global imaged volume (Fig. 3h), which increased throughout gastrulation and plateaued at the end of gastrulation (10 hpf). It is also consistent with an estimate of the average proliferation rate during the same developmental period[3]. (d) Average number of neighbors as a function of hpf. In both the zebrafish and the ascidian, this value is near 12, i.e. in the range of the perfect 3D hexagonal close packing[4]. In the sea urchin, the observed value of about 10 neighbors is consistent with the presence of a blastocoel and the organization of its early embryo into a pseudostratified epithelium. (e) Dispersion of cells as a function of hpf. At each time step, all possible pairs of neighboring cells are identified and tracked forward. Distances between neighbors are divided by the average internuclear distance. The resulting average normalized distance increases over time, providing a quantitative evaluation of cell dispersion. Information about the global cohesiveness of the embryonic tissues and indicates distinct developmental periods.

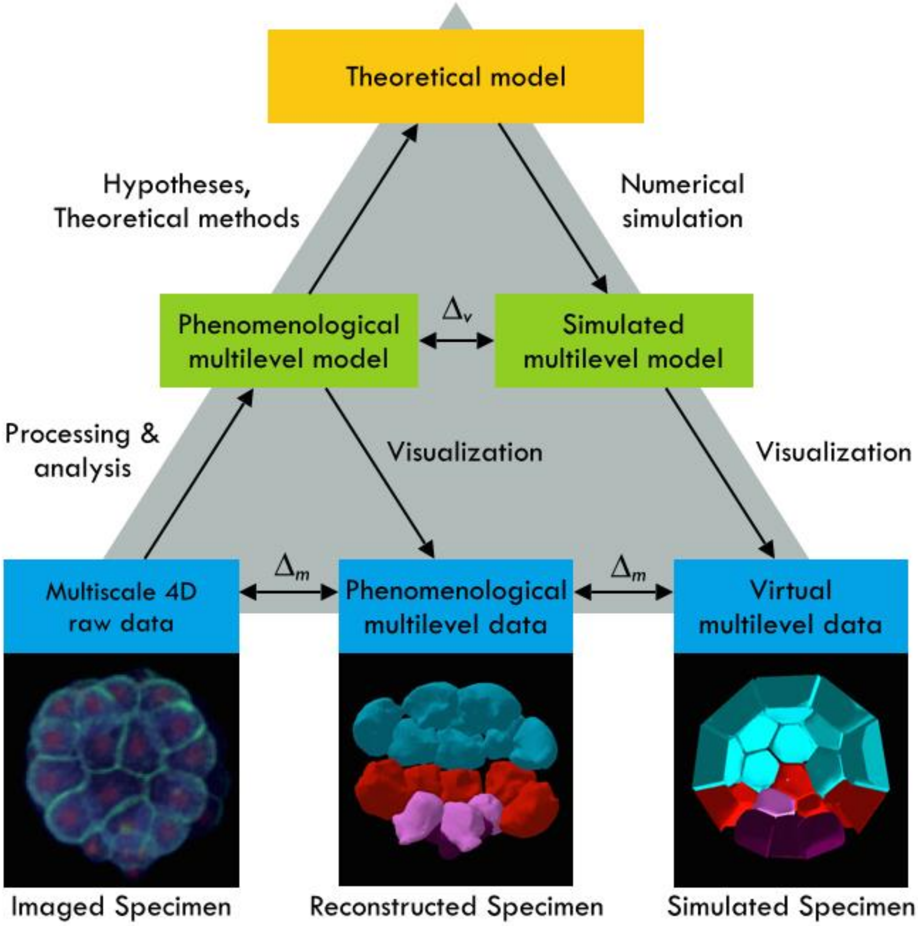

Supplementary Figure 4 | Formal and applied epistemology for the reconstruction of the multilevel dynamics of complex systems. This diagram summarizes our general methodology for the integrative modeling of living systems' morphogenesis. Ultimately, imaging data revealing the characteristic scales of biological processes should lead to models coupling the different organization levels of developing embryos (molecular, cellular and tissular). The methods and tools provided in this publication are specific to the cellular level of organization. The architecture of the workflow accessible through our webservice, however, is ready for the integration of new algorithms operating on other types of data. The three bottom pictures illustrate our concepts with the biomechanical modeling of the sea urchin early embryogenesis (blastula stages): *imaged live specimen* (bottom-left panel), *reconstructed specimen* (bottom-center panel), and *simulated specimen* with the MecaGen platform[5] (bottom-right panel, image courtesy of Julien Delile). We call *phenomenological multilevel model*, or "reconstruction" (middle-left panel and underlying triangle graph) the set of operations based on algorithmic methods to extract measurements from 3D+time images and provide the most accurate quantitative information relevant to the detected components: cell numbers, positions, shapes, interactions, trajectories, and so on. The design and implementation of image processing algorithms, data management, and analysis tools are key steps toward high-throughput 3D+time image analysis. This phenomenological reconstruction feeds a database of raw and reconstructed data used for statistical analysis. Quantitative results are then used to set the parameters of a *theoretical model* (top panel) and its derived numerical simulations, leading to a *simulated multilevel model*, or "virtual morphogenesis" (middle-right panel). This scheme creates a virtuous cycle of experimental validation of the models (top triangle graph) by questioning both the biological system and the simulation. Measuring the differences (Δ's) between the *multiscale raw data* (imaged specimen), the *phenomenological data* (reconstructed specimen) and the *virtual data* (simulated specimen), constitutes a major challenge. So far, the Δ between the raw and reconstructed data is estimated by a manual procedure using our custom-made visualization interface Mov-IT.

Supplementary Table 1 | Comparative performance of BioEmergences and eight software tools on dataset Dr1

	Lineage Score	Center Detection Rates			Linkage Rates only on TP centers with a GS past link			Mitosis Detection Rates only on TP centers		
**Software Tool**	%Sensitivity of centers × %Sensitivity of links	%Sensitivity: TP/(TP+FN)[a]	%False Detection: FP/(TP+FP)	%False Negative: FN/(TP+FN)	%Sensitivity: TP/(TP+FN)	%False Linkage: FP/(TP+FP)	%False Negative: FN/(TP+FN)	%Sensitivity: TP/(TP+FN)	%False Detection: FP/(TP+FP)	%False Negative: FN/(TP+FN)
BioEmergences Workflow	**96.04%**	98.11%	0.21%	1.89%	97.89%	1.03%	2.11%	67.37%	37.03%	32.63%
Imaris Autoregressive Motion Expert	**15.50%**	30.38%	2.22%	69.62%	51.03%	42.43%	48.97%	0.00%	N/A	100.00%
Imaris Autoregressive Motion	**27.40%**	35.08%	0.25%	64.92%	78.10%	20.71%	21.90%	0.00%	N/A	100.00%
Imaris Brownian Motion	**29.43%**	36.22%	0.24%	64.14%	82.05%	17.02%	17.95%	0.00%	N/A	100.00%
Imaris Connected Component	**3.33%**	32.34%	0.01%	67.66%	10.31%	8.60%	89.69%	0.00%	N/A	100.00%
Icy Spot Tracking	**41.42%**	47.35%	0.39%	52.65%	87.49%	9.72%	12.51%	0.00%	N/A	100.00%
Volocity Shortest Path	**17.80%**	31.04%	44.25%	68.96%	57.33%	39.26%	42.67%	0.00%	N/A	100.00%
Volocity Trajectory Variation	**12.82%**	31.07%	44.44%	68.93%	41.27%	43.02%	58.73%	0.00%	N/A	100.00%
Amat et al. 2014	**83.06%**	87.45%	0.86%	12.55%	94.99%	4.06%	5.01%	12.93%	82.06%	87.07%

	Mitosis Detection Counts only on TP centers															
**Software Tool**	#Gold Std = GS				#True Pos = TP				#False Pos = FP				#False Neg = FN			
	*t = 4.36**	*t = 6.22*	*t = 8.08*	*t = 9.95*	*t = 4.36*	*t = 6.22*	*t = 8.08*	*t = 9.95*	*t = 4.36*	*t = 6.22*	*t = 8.08*	*t = 9.95*	*t = 4.36*	*t = 6.22*	*t = 8.08*	*t = 9.95*
BioEmergences Workflow					203	22	12	15	6	7	17	25	53	4	12	12
Imaris Autoregressive Motion Expert					0	0	0	0	0	0	0	0	37	7	8	4
Imaris Autoregressive Motion					0	0	0	0	0	0	0	0	153	10	9	6
Imaris Brownian Motion					0	0	0	0	0	0	0	0	162	11	10	7
Imaris Connected Component	264	26	24	27	0	0	0	0	0	0	0	0	170	5	8	4
Icy Spot Tracking					0	0	0	0	0	0	0	0	197	14	13	4
Volocity Shortest Path					0	0	0	0	0	0	0	0	52	3	4	4
Volocity Trajectory Variation					0	0	0	0	0	0	0	0	51	3	4	4
Amat et al. 2014					83	2	1	0	49	23	111	116	135	20	21	23

Software Tool	Center Detection Counts															
	#Gold Std = GS				#True Pos = TP				#False Pos = FP				#False Neg = FN			
	*t = 4.36**	*t = 6.22*	*t = 8.08*	*t = 9.95*	*t = 4.36*	*t = 6.22*	*t = 8.08*	*t = 9.95*	*t = 4.36*	*t = 6.22*	*t = 8.08*	*t = 9.95*	*t = 4.36*	*t = 6.22*	*t = 8.08*	*t = 9.95*
BioEmergences Workflow					4292	4035	10600	7262	15	1	25	18	64	25	357	164
Imaris Autoregressive Motion Expert					1547	1682	2700	1479	16	67	51	33	2809	2378	8257	5947
Imaris Autoregressive Motion					2226	1751	2797	1528	3	11	7	0	2130	2309	8160	5898
Imaris Brownian Motion					2322	1791	2793	1525	4	3	1	9	2034	2269	8164	5901
Imaris Connected Component	4356	4060	10957	7426	2427	1448	2298	1263	1	0	0	0	1929	2612	8659	6163
Icy Spot Tracking					2849	2048	4717	2264	26	3	15	4	1507	2012	6240	5162
Volocity Shortest Path					1936	946	2871	2244	1156	1096	2192	1669	2420	3114	8086	5182
Volocity Trajectory Variation					1930	953	2869	2252	1184	1098	2231	1663	2426	3107	8088	5174
Amat et al. 2014					3748	3926	9730	5810	43	22	42	78	608	134	1227	1616

Software Tool	Linkage Counts<br>only on TP centers with a GS past link															
	#Gold Std = GS				#True Pos = TP				#False Pos = FP = WL<br>#Wrong Links (WL)[b]				#False Neg = FN = WL + ML<br>#Missing Links (ML)[c]			
	*t = 4.36**	*t = 6.22*	*t = 8.08*	*t = 9.95*	*t = 4.36*	*t = 6.22*	*t = 8.08*	*t = 9.95*	*t = 4.36*	*t = 6.22*	*t = 8.08*	*t = 9.95*	*t = 4.36*	*t = 6.22*	*t = 8.08*	*t = 9.95*
BioEmergences Workflow					3079	3641	9548	6501	44<br>44	15<br>15	98<br>98	84<br>84	132<br>88	26<br>11	171<br>73	123<br>39
Imaris Autoregressive Motion Expert					356	715	858	518	145<br>145	571<br>571	939<br>939	409<br>409	284<br>139	773<br>202	1067<br>128	409<br>0
Imaris Autoregressive Motion					1075	1167	1426	805	100<br>100	288<br>288	606<br>606	264<br>264	119<br>19	341<br>53	621<br>15	264<br>0
Imaris Brownian Motion					1170	1258	1559	847	101<br>101	239<br>239	478<br>478	221<br>221	112<br>11	283<br>44	492<br>14	221<br>0
Imaris Connected Component	3329	3841	10283	7029	103	94	97	167	2<br>2	9<br>9	23<br>23	8<br>8	1273<br>1271	1100<br>1091	1502<br>1479	676<br>668
Icy Spot Tracking					1456	1566	3482	1632	136<br>136	170<br>170	481<br>481	150<br>150	343<br>207	182<br>12	481<br>0	150<br>0
Volocity Shortest Path					503	446	1235	920	336<br>336	223<br>223	782<br>782	749<br>749	389<br>53	256<br>33	921<br>139	845<br>96
Volocity Trajectory Variation					399	299	890	647	272<br>272	205<br>205	672<br>672	594<br>594	486<br>214	409<br>204	1264<br>592	1128<br>534
Amat et al. 2014					2543	3510	8495	4754	84<br>84	54<br>54	344<br>344	394<br>394	141<br>57	66<br>12	378<br>34	452<br>58

**times in hours post fertilization (hpf) – [a]displayed rates are averages of four ratios, one per time interval centered in 4.36 hpf, 6.22 hpf, 8.08 hpf and 9.95 hpf*
[b]wrong links, which connect a cell to a wrong target, contribute both to false positives (by creating new links that do not exist) and to false negatives (by missing the correct links)
[c]missing links, which correspond to a cell without any link, contribute only to false negatives

Supplementary Table 2 | Developmental table of the imaged embryos (3 species, 2 specimens for each species)

	Danio rerio						*Phallusia mammillata*						*Paracentrotus lividus*					
dataset	Dr1 (Δt = 67 sec)			Dr2 (Δt = 153 sec)			Pm1 (Δt = 180 sec)			Pm2 (Δt = 180 sec)			Pl1 (Δt = 207 sec)			Pl2 (Δt = 180 sec)		
TS	ID	TDT	DS	ID	TDT	DS	ID	TDT	DS	ID	TDT	DS	ID	TDT	DS	ID	TDT	DS
0	0.00	4.17	*late sphere*	0.00	5.40	*50% epiboly*	0.00	5.00	*initial gastrula*	0.00	3.00	*35-cell*	0.00	4.33	*32-cell*	0.00	4.33	*32-cell*
10	0.19	4.35		0.43	5.83		0.50	5.50		0.50	3.50		0.58	4.91		0.50	4.83	
20	0.37	4.54		0.85	6.25	*shield*	1.00	6.00		1.00	4.00		1.15	5.48		1.00	5.33	
30	0.56	4.73		1.28	6.68		1.50	6.50		1.50	4.50		1.73	6.06		1.50	5.83	
40	0.74	4.91		1.70	7.10		2.00	7.00		2.00	5.00		2.30	6.63		2.00	6.33	
50	0.93	5.10		2.13	7.53		2.50	7.50		2.50	5.50		2.88	7.21		2.50	6.83	
60	1.12	5.28		2.55	7.95		3.00	8.00		3.00	6.00		3.45	7.78		3.00	7.33	
70	1.30	5.47		2.98	8.38	*75% epiboly*	3.50	8.50	*early tail bud II*	3.50	6.50		4.03	8.36		3.50	7.83	
80	1.49	5.66		3.40	8.80					4.00	7.00		4.60	8.93		4.00	8.33	
90	1.68	5.84		3.83	9.23					4.50	7.50		5.18	9.51	*h.blastula*	4.50	8.83	
100	1.86	6.03		4.25	9.65					5.00	8.00					5.00	9.33	
110	2.05	6.21		4.68	10.08	*tail bud*				5.50	8.50					5.50	9.83	
120	2.23	6.40	*Late shield*	5.10	10.50					6.00	9.00	*early tail bud II*				6.00	10.33	*h.blastula*
150	2.79	6.96		6.38	11.78													
200	3.72	7.89		8.50	13.90	*8-somite*												
250	4.65	8.82																
300	5.58	9.75																
320	5.95	10.12	*Tail bud*															
360	6.70	10.87	*1-somite*															

TS : Time Step
ID : Imaging Duration (in hours)
TDT : Total Development Time (in hours post fertilization, hpf)
DS : Developmental Stage

Developmental stages for the different species as described in (*Danio rerio*)[6], (*Ciona intestinalis*)[7] and (*Strongylocentrotus sp.)*[8].

Supplementary Table 3 | Description of parameters

**Parameters for the GMCF filtering method***				
Name	Description	Useful range	Used values	For noisy data
**K**	parameter in the "diffusivity" function *g*; a larger **K** means that edges are better respected (make stronger obstacles) and thus better preserved by the nonlinear diffusion process	0.1 – 100	5.0	2.5
**τ**	time step in the discretization of the nonlinear diffusion model; a larger **τ** means that more smoothing is applied in one time step	1 – 1.0e-4	2.0e-4	0.0016
**σ**	time step for the linear diffusion used to pre-smooth the image gradient (edge detector) in the "diffusivity" function *g*; a larger **σ** means more smoothing of the gradient inside the edge detector, thus the final result is less sensitive to noise	1 – 1.0e-4	1.0e-4	0.0001
**ε**	regularization parameter inside the GMCF model used to prevent zero gradients in the denominators of the numerical scheme	1 – 1.0e-6	1.0e-4	1.0e-4
**iter**	number of time steps; a small **iter** means less smoothing of the image; a larger **iter** means more smoothing	5 – 15 depending on **τ**	5 for Dr1 nuclei, 10 for all others	15

**A more detailed explanation of the method, the meaning and choice of the parameters can be found in[9].*

**Parameters for the FBLS center detection method****				
Name	Description	Useful range	Used values	For noisy data
**F**	speed of advection in the normal direction	1-5	1	1.5
**D**	strength of the mean curvature flow diffusion	1 – 1.0e-4	12.5e-4	0.003
**epsilonD**	regularization parameter in the advective part of the FBLS model used to prevent zero gradients in the denominators of the numerical scheme	1-1.0e-6	1	1.0
**epsilonF**	regularization parameter in the mean curvature part of the FBLS model used to prevent zero gradients in the denominators of the numerical scheme	1 – 1.0e-6	1.0e-6	1.0e-6
**τ**	time step of the FBLS center detection method; **τ** is restricted by the Courant-Friedrichs-Lewy (CFL) stability condition	5 – 1.0e-4	12.5e-4 for Dr2, 5e-4 for others	0.001
**threshold**	the local maxima above this threshold are counted as cell centers at the current time step; a small **threshold** means that more centers (even inside one cell) are detected; a large **threshold** means that less centers are detected	1 – 1.0e-2	8.0e-2	0.06
**iter**	number of time steps	4 – 50 depending on **τ**	15 (Dr1) 4 (Dr2) 10 (Pl1) 16 (Pl2), 30 (Pm)	40

***A more detailed explanation of the method, the meaning and choice of the parameters can be found in[10,11].*

Parameters for the DoG center detection algorithm								
Name	Description	Useful range	Used values (first → last time step)					
			Dr1	Dr2	Pl1	Pl2	Pm1	Pm2
**Std Small**	standard deviation of one Gaussian in μm, related to the smallest possible nucleus size	1 – 3.1	2.4 → 1.6	2.4 → 2.2	2.2 → 1.2	1.8 → 1.2	2.8 → 1.6	2.6 → 1.6
**Std Big**	standard deviation of one Gaussian in μm, related to the largest possible nucleus size	10 – 20	16	18	12	12 → 14	12 → 16	12 → 18
**Threshold**	normalized threshold of signal intensity	1 – 9e-2	3 → 4e-2	4 → 3e-2	2 → 4e-2	2 → 4e-2	3e-2	1e-2

Parameters for the Simulated Annealing (SimAnn) cell tracking algorithm								
Name	Description	Useful range	Used values					
			Dr1	Dr2	Pl1	Pl2	Pm1	Pm2
$\mathbf{p_1}$	proportion of cells where SimAnn is applied; cells are sorted by decreasing cost and only the ones with the highest costs are processed	0.2 – 1	0.5	0.5	0.4	0.5	0.5	0.4
$\mathbf{p_2}$	number of repetitions in the entire SimAnn run	1 – 10	3	3	3	4	3	3
$\mathbf{p_3}$	number of nearest cells selected to modify the links from a given cell	1 – 5	1	2	3	2	2	2
$\mathbf{p_4}$	number of iterations by cell: if *n* cells are being processed, SimAnn will perform $n\times\mathbf{p_4}$ tentative link modifications	1 – 20	11	10	10	9	10	11
$\mathbf{p_5}$	the initial temperature *T* is computed to make the probability of accepting a move (when the cost increases by an amount equal to the average of the 100 highest costs in the population) equal to $\mathbf{p_5}$.	0 – 0.3	0.1	0.1	0	0.1	0.1	0.1
$\mathbf{p_6}$	same as p_5 in determining the final temperature	0 – 0.01	1e-3	1e-3	1e-3	0	1e-3	1e-3
$\mathbf{p_7}$	coefficient of the cost associated to the deformation of the tissue between times *t* and *t+1* (this deformation depends on the choice of links)	1 – 3	1	1	1	1	1	1
$\mathbf{p_8}$	coefficient of the cost associated with enforcing symmetric behaviors of daughter cells	0 – 10	5	5	5	4	3	5
$\mathbf{p_9}$	coefficient of the cost associated with enforcing some amount of inertia (to cope with noise)	0 – 10	1	1	1	1	1	1
$\mathbf{p_{10}}$	coefficient of the cost penalizing the end of a lineage branch (i.e. the disappearance of a cell)	100 – 200	190	200	190	200	200	200
$\mathbf{p_{11}}$	coefficient of the cost penalizing the sudden appearance of a cell out of any former lineage	100 – 200	140	150	160	150	150	150
$\mathbf{p_{12}}$	coefficient of the cost penalizing divisions occurring too early after a previous division	0 – 10	2	3	2	2	2	2
$\mathbf{p_{13}}$	coefficient of the cost penalizing acceleration	0 – 7	2	2	2	1	2	3
$\mathbf{p_{14}}$	coefficient of the cost penalizing speeds above a certain threshold (the threshold is hardcoded)	0 – 10	3	4	2	3	3	4
$\mathbf{p_{15}}$	coefficient of the cost penalizing a cell at time *t+1* not linked to its closest neighbor at time *t*	0 – 50	20	20	15	20	20	20
$\mathbf{p_{16}}$	coefficient favoring sisterhood of simultaneously born cells; $\mathbf{p_{16}}$ is used only when external information about division is available	0 – 50	10	15	15	15	15	15

Parameters for the nucleus and membrane segmentation SubSurf method***[a]			
Name	Description	Useful range	Used values
**K**	parameter in the edge detection function *g*; a larger **K** means that edges (but also noisy structures) are better respected by the segmentation process	1 – 10e+3	1e+3
**V_adv**	speed of advection in the SubSurf model	1 – 20	10
**V_curv**	strength of the mean curvature flow regularization in the SubSurf model	0.1 – 5	0.2
**τ**	time step for the segmentation method; **τ** is restricted by the Courant-Friedrichs-Lewy (CFL) stability condition in the advective part of the SubSurf model and should not be larger than 1/**V_adv**	0.05 – 1	0.1
**σ**	time step for the linear diffusion used to pre-smooth the segmented image gradient (edge detector); a larger **σ** means more smoothing of the gradient inside the edge detector, thus the final result is less sensitive to noise but it can be oversmoothed as well	1 – 20e-4	1e-4
**epsilon**	regularization parameter to prevent zero gradients in the denominators of the numerical scheme	1e-6	1e-6
**edge_power**	parameter inside the function *g*; the norm of gradient is raised to the power **edge_power**	1 – 6	1
**convolutionU**	if equal to 1, the linear diffusion smoothing (convolution) with parameter **σ** is applied to the segmented image; if equal to 0, the linear diffusion is not applied	0 or 1	1
**convolutionPMC**	if equal to 1, the linear diffusion smoothing (convolution) with parameter **σ** is applied to the pixelwise output of the edge detection function *g*; if equal to 0, such linear diffusion is not applied	0 or 1	0
**iter**	number of time steps in the segmentation process	200 – 500 for nuclei, 1000 – 2000 for membranes	250 for nuclei, 1200 for membranes
**embryo_type**	if equal to 1, the method is tuned for small and packed cells (i.e. Dr1-2 datasets); if equal to 2, then it can be used for large cells (i.e. Pl1-2 and Pm1-2 datasets) [b]	1 or 2	1 for Dr1, Dr2, 2 for the others

****A more detailed explanation of the method, the meaning and choice of the parameters can be found in*[11,12].

[a]*The result of the segmentation process is in the form of VTK files containing integer values between 0 and 255 in every voxel. The segmented object can be rendered as triangulated surface by choosing a suitable isosurface value in the resulting VTK file (usually 128, but other values should be tried to obtain the best fit with the raw data). By choosing a larger isosurface value, one can obtain smaller segmented objects; by choosing a smaller isosurface value, larger segmented objects.*

[b]*The choice of 1 or 2 for* ***embryo_type*** *is related to the initial condition of the SubSurf segmentation process. The usual procedure is to construct a certain initial shape around the cell center, which is then evolved by the SubSurf method. This initial condition should be chosen according to the size of the cells and their expected shape. For different types of data and species, one can either try choices 1 or 2 or then customize the code of the procedure used to construct the initial condition. For user support, please contact: mikula@math.sk*

Supplementary Note 1 | Computational speed and scalability. Typical image datasets processed here contain 512×512×120 voxels and 2 channels (e.g. Dr2 dataset). Approximate computational cost of the algorithms:

- The filtering step with the GMCF method and 10 iterations takes 5 minutes for each 3D volume using 8 processors (communicating by MPI).
- The cell center detection step by FBLS runs for 20 iterations and takes 200 seconds for one 3D volume using 8 processors (communicating by MPI).
- The cell center detection step by DoG (combining filtering and detection processes) takes on average 5.5 seconds per time step, while time steps can be processed in parallel on several processors.
- For nucleus segmentation using SubSurf, it takes on average 0.75 second to process each nucleus. With 6,000 nuclei, a single 3D volume is segmented in 1.25 hours
- The computation time for the membrane segmentation is 6 seconds per cell (10 hours for a 3D volume containing 6,000 cells).
- Whole embryo shape segmentation can also be operated in parallel.
- The cell-tracking algorithm SimAnn takes 4 hours on 8 processors to process a dataset with 360 time steps and an average of 6,000 cells per 3D volume.

In sum, reconstructing the cell lineage tree (e.g. with DoG and SimAnn) from a typical zebrafish dataset (512×512×120 voxels with in average 6,000 cells over 360 time steps) with the standalone version of the BioEmergences workflow takes less than 5 hours on a local computer with 8 cores. Performing the complete reconstruction (e.g. lineage tree and shapes from a two channels dataset) in the web service mode with computation on EGI (European Grid Infrastructure) can take 48 hours including data transfer and job queuing until execution. But it should be noted that a number of datasets can be processed in parallel during this period.

Supplementary Note 2 | *In silico* fate mapping can be performed in three different ways. For the ascidian embryos, the state-of-the-art fate map proposed at the 110-cell stage[13] is implemented by defining distinct cell populations with the Mov-IT visualization software, and assigning them specific colors. The fate map is then propagated along the reconstructed cell lineage. It is this propagation across cohorts of specimens that can tell us whether the lineage is invariant, as it is traditionally assumed. It should be noted that a similar strategy led our colleagues working with *C. elegans* to revise their ideas about lineage invariance in this species[14].

Alternatively, and without a priori, cell fate can be assessed as in classical embryology studies, i.e. by following cell clonal history long enough to be able to conclude about the contribution of progenitors to organs, and also about cell differentiation in specific cell types defined by their shape, position and neighborhood. The limitation here is the duration of the time lapse and the evolution of image quality, hence of tracking accuracy. We now know that beyond 15 hpf, it becomes very difficult to resolve individual nuclei in ubiquitously stained zebrafish embryos, even when zooming in on a specific compartment. In this case, mosaic or rainbow type staining is required to decrease image complexity and improve tracking accuracy. The methods provided here are expected to perform well at any stage of development with mosaic staining of nuclei.

Finally, there is also the possibility to backtrack cells from their location at a late stage when compartments or presumptive organs are morphologically recognizable. This is achieved with Mov-It by using the "cell selection" function and back-propagating along the cell tracking.

Supplementary References

1. Amat, F. *et al.* Fast, accurate reconstruction of cell lineages from large-scale fluorescence microscopy data. *Nat Methods* **11**, 951–958 (2014).
2. Pion, S. & Teillaud, M. 3D Triangulations. CGAL User and Reference Manual. *CGAL Editorial Board, 4.5 Edition,* (2014).
3. Kane, D., Warga, R. & Kimmel, C. Mitotic domains in the early embryo of the zebrafish. *Nature* (1992).
4. Farhadifar, R., Röper, J. C., Aigouy, B., Eaton, S. & Jülicher, F. The influence of cell mechanics, cell-cell interactions, and proliferation on epithelial packing. *Current Biology* **17**, 2095–2104 (2007).
5. Delile, J., Doursat, R., Peyrieras, N. & Kriete, A. Computational modeling and simulation of animal early embryogenesis with the mecagen platform. *... Systems Biology* (2013).
6. Kimmel, C. B., Ballard, W. W., Kimmel, S. R., Ullmann, B. & Schilling, T. F. Stages of embryonic development of the zebrafish. *Dev Dyn* **203**, 253–310 (1995).
7. Hotta, K. *et al.* A web-based interactive developmental table for the ascidian Ciona intestinalis, including 3D real-image embryo reconstructions: I. From fertilized egg to hatching larva. *Dev Dyn* **236**, 1790–1805 (2007).
8. Stephens, R. E. Studies on the development of the sea urchin Strongylocentrotus droebachiensis. I. Ecology and normal development. *The Biological Bulletin* **142**, 132–144 (1972).
9. Kriva, Z., Mikula, K., Peyrieras, N., Rizzi, B., Sarti, A., Stasova, O. 3D Early Embryogenesis Image Filtering by Nonlinear Partial Differential Equations. *Medical Image Analysis* **14**(4), 510-526 (2010).
10. Frolkovic, P., Mikula, K., Peyrieras, N., Sarti, A. A counting number of cells and cell segmentation using advection-diffusion equations, *Kybernetika* **43**(6), 817-829 (2007)
11. Bourgine, P., Cunderlik, R., Drblikova, O., Mikula, K., Peyrieras, N., Remesikova, M., Rizzi, B., Sarti, A. 4D embryogenesis image analysis using PDE methods of image processing, *Kybernetika* **46**(2) 226-259 (2010).

12 Mikula, K., Peyrieras, N., Remesikova, M., Stasova, O. Segmentation of 3D cell membrane images by PDE methods and its applications. *Computers in Biology and Medicine* 41(6), 326-339 (2011).

13. Lemaire, P. Unfolding a chordate developmental program, one cell at a time: invariant cell lineages, short-range inductions and evolutionary plasticity in ascidians. *Dev Biol* **332**, 48–60 (2009).
14. Bao, Z. *et al.* Automated cell lineage tracing in Caenorhabditis elegans. *Proc. Natl. Acad. Sci. U.S.A.* **103**, 2707–2712 (2006).
